# ﻿Reconsideration of some populations of *Euscorpiusconcinnus* complex (Scorpiones, Euscorpiidae)

**DOI:** 10.3897/zookeys.1100.78979

**Published:** 2022-05-16

**Authors:** Gioele Tropea, Aristeidis Parmakelis

**Affiliations:** 1 Via Gavinana 2, 00192 Rome, Italy Unaffiliated Rome Italy; 2 Section of Ecology and Taxonomy, Department of Biology, National and Kapodistrian University of Athens, Panepistimioupoli Zografou, GR-15772, Athens, Greece National and Kapodistrian University of Athens Athens Greece

**Keywords:** In the present work, several scorpion populations assigned to *Euscorpiusconcinnus* (C.L. Koch, 1837) and *Euscorpiuscarpathicusniciensis* (C.L. Koch, 1841) are reconsidered on a phylogenetic, morphological, and geographical basis. Three new species are described, *E.latinus* **sp. nov.**, *E.stefaniae* **sp. nov.**, and *E.trejaensis***sp. nov.**, while *E.niciensis***stat. nov.** is elevated to species status. Ecological and biogeographical data are provided for the revised taxa. Following these taxonomic changes, the number of species comprising the subfamily Euscorpiinae has increased to 90. The scorpion species present in Italy increased to 27, with one species belonging to the family Buthidae, one species to Belisariidae, and 25 species to Euscorpiidae. Euscorpiinae, *Euscorpius*, France, Italy, new species, phylogenetic, scorpion

## ﻿Introduction

*Scorpiusconcinnus* was described by C.L. Koch in 1837 from an unknown locality. Since then, the first real reassessment of this species was published by the Italian arachnologist Lodovico Di Caporiacco (1950), in a revision that treated the species and subspecies present in Italy and in some neighbouring countries. Di Caporiacco downgraded *Scorpiusconcinnus* to subspecies of *Euscorpiuscarpathicus* (L., 1767), identifying it as the most common subspecies in Italy, with a range extending from Tuscany to Campania. Fet and Soleglad (2002) did not consider it a valid taxon and synonymised it with *Euscorpiustergestinus* (C.L. Koch, 1837) along with other taxa having the character eb = 4, including *Euscorpiuscarpathicusniciensis* (C.L. Koch, 1841). Subsequently, Vignoli et al. (2005) compared some populations assigned to *Euscorpiuscarpathicusconcinnus* sensu Di Caporiacco (1950) with typical specimens of *E.tergestinus* sensu Fet and Soleglad (2002) (which, as we now know, were *Euscorpiusaquilejensis* (C.L. Koch, 1837) (Tropea 2013)) coming to the conclusion that they were two distinct species, and elevated *E.concinnus* to species status, designating the neotype from Siena (Tuscany, Italy). Salomone et al. (2007) presented some phylogenetic reconstructions, which already identified several supported clades within the *E.concinnus* group, well correlated with geographic sampling. The taxon *E.c.niciensis* has not been treated by Vignoli et al. (2005) and therefore officially remained as *E.tergestinus* until the work of Tropea (2013), who redescribed *E.tergestinus*, did not accept the synonymy of Fet and Soleglad (2002) and stated that *E.c.niciensis* is morphologically and phylogenetically relatively close to *E.concinnus*. Since then, the status of this taxon has been awaiting clarification, although some authors have considered specimens collected in France as belonging to *E.concinnus* (see Ythier 2011; Graham et al. 2012), but without justifying the assignment.

In the present work, populations assigned to *E.concinnus* and *E.c.niciensis* are reconsidered on a phylogenetic, morphological and geographical basis, elevating *E.niciensis* stat. nov. to species status and three new species of the *E.concinnus* complex are described: *Euscorpiuslatinus* sp. nov., *Euscorpiusstefaniae* sp. nov., and *Euscorpiustrejaensis* sp. nov. Following these taxonomic changes, the number of scorpion species in Italy has increased to 27, of which one species belongs to the family Buthidae, one species to Belisariidae, and 25 species to Euscorpiidae, and the number of Euscorpiinae species in the world has increased to 90.

## ﻿Materials and methods

A total of 365 specimens of the *E.concinnus* complex has been examined from Italy (Piedmont, Liguria, Toscana, Emilia Romagna, Veneto, Lazio and Molise) and France. A detailed list of the specimens with label data is provided under each species description. Most of the specimens were collected by the first author from 2009 to 2020. The specimens were found under stones, bricks, tree branches, and trunks (bark and cracks of trees were also investigated). In addition, specimens were searched for during night time using a UV light flashed against the surfaces, spaces and cracks of dry-stone walls and houses. The specimens were preserved in 80% or 96% ethanol, at room temperature or -21 °C, while some specimens were examined in situ and released by taking preliminary data such as sex, pectinal teeth number, trichobothria on the pedipalps patella ventral surface, measurement of carapace, metasoma, telson, and total length. We also examined several specimens from different museum collections (see Depositories list), and those donated by colleagues, friends, relatives and enthusiasts. Geographical coordinate data are in decimal degrees and were recorded with a portable GPS device.

The trichobothrial notation follows Vachon (1974). Morphological measurements (given in mm) and abbreviations follow Tropea et al. (2014), but we use **Wchel = WchelA**. Morphological nomenclature follows Stahnke (1971), Hjelle (1990) and Sissom (1990); the chela carinae and denticle configuration follows Soleglad and Sissom (2001) but we treated **ID** plus **IAD** as a single character (**ID**). Hemispermatophore nomenclature follow Molteni et al. (1983), Fet and Soleglad (2002) and Tropea (2021).

### ﻿Abbreviations

**CarA-CarP** % distances from centre of median eyes to anterior and posterior margins of the carapace;

**Dp** pectinal teeth number;

**H** height;

**Htel** telson height;

**juv.** juvenile (immature specimen in any stage of development);

**L** length;

**lb** basal lobe;

**Lcar** carapace length;

**Lchel** chela length;

**lde** external distal lobe;

**ldi** internal distal lobe;

**Lfem** femur length;

**Lpat** patella length;

**Ltel** telson length;

**Lmet** sum of the length of all metasomal segments;

**met.seg** metasomal segment;

**NCS** specimens examined in nature and released;

**Pe** trichobothria on pedipalp patella external surface;

**Pv**trichobothria on the pedipalp patella ventral surface;

**Wcar**carapace width;

**Wchel** chela width (= WchelA of Tropea et al. 2014);

**Wmet** sum of the width of all metasomal segments.

### ﻿Depositories

**CNBFVR** Corpo Forestale dello Stato: Centro nazionale per lo studio e la conservazione della biodiversità forestale, Verona;

**GTC** personal collection of Gioele Tropea, Rome, Italy;

**MSNB** Museo Civico di Scienze Naturali E. Caffi, Bergamo, Italy;

**MSNV** Museo di Storia Naturale di Verona, Italy;

**MZUF**Museo di Storia Naturale dell’Università degli Studi di Firenze, sezione di Zoologia La Specola, Florence, Italy;

**MZUR**Museo di Zoologia dell’Università di Roma La Sapienza, Rome, Italy;

**VVZC** Collection of V. Vignoli, Dipartimento di Biologia Evolutiva, University of Siena, Italy.

### ﻿Sequence data and phylogenetic analyses

For this study, we extracted DNA from 14 specimens. The DNA extraction protocol applied is described in Parmakelis et al. (2013). Sequence data were generated for the 16S rDNA and COX1 mtDNA loci. More specifically we generated ten sequences of 16S rDNA and 14 sequences of COX1. The PCR protocols implemented and primers used are as reported in Parmakelis et al. (2013). All PCR generated amplicons were purified using a commercial kit (Macherey-Nagel) before being sequenced. Automated sequencing of both strands of amplicons was performed using Big-Dye terminator chemistry. The primers used in the sequencing reactions were the same as in the PCR amplifications.

**Table 1.** Measurements (mm) of *Euscorpiusconcinnus*, *E.latinus* sp. nov., and *E.niciensis* stat. nov.

		*E.concinnus*		*E.latinus* sp. nov.		*E.niciensis* stat. nov.	
		Topotype ♂	Topotype ♀	Holotype ♂	Paratype ♀	Neotype ♂	Topotype ♀
**Total**	**Length**	28.26	30.18	27.30	27.58	33.08	33.43
**Carapace**	**Length**	4.05	4.60	4.20	4.20	4.80	5.10
	**Post. width**	4.10	4.80	4.30	4.40	5.20	5.40
**Metasoma**	**Length**	10.66	11.00	11.10	9.89	12.53	12.03
**Segment I**	**Length**	1.38	1.45	1.40	1.25	1.60	1.60
	**Width**	1.68	1.60	1.59	1.57	1.80	1.75
**Segment II**	**Length**	1.59	1.65	1.70	1.50	1.90	1.80
	**Width**	1.50	1.45	1.48	1.40	1.62	1.60
**Segment III**	**Length**	1.78	1.90	1.90	1.70	2.10	2.05
	**Width**	1.42	1.36	1.40	1.30	1.60	1.50
**Segment IV**	**Length**	2.21	2.30	2.30	2.04	2.55	2.48
	**Width**	1.40	1.28	1.35	1.25	1.50	1.40
**Segment V**	**Length**	3.70	3.70	3.80	3.40	4.38	4.10
	**Width**	1.38	1.22	1.35	1.25	1.56	1.40
**Telson**	**Length**	3.95	3.78	4.30	3.49	4.75	4.30
**Vesicle**	**Length**	2.80	2.48	3.10	2.29	3.70	2.80
	**Width**	1.70	1.29	1.55	1.20	1.91	1.40
	**Height**	1.80	1.30	1.80	1.13	2.10	1.30
**Aculeus**	**Length**	1.15	1.30	1.20	1.20	1.05	1.50
**Femur**	**Length**	3.40	3.80	3.40	3.30	4.00	4.12
	**Width**	1.30	1.50	1.30	1.30	1.60	1.60
**Patella**	**Length**	3.38	3.84	3.48	3.45	4.10	4.25
	**Width**	1.41	1.65	1.45	1.60	1.80	1.94
**Chela**	**Length**	7.00	7.98	7.10	7.00	8.38	8.82
	**Width**–**A**	2.80	3.05	2.88	2.70	3.60	3.50
**Movable finger**	**Length**	4.15	4.65	4.20	3.80	5.20	5.30

For each locus, generated sequences (forward and reverse strands) were assembled (built-in algorithm), edited and aligned using CodonCode aligner v.2.0.6. The ClustalW algorithm was implemented in the alignment process. The aligned dataset of 16S rDNA was comprised of 13 sequences and was 435 bp in length. The COX1 aligned dataset included 15 sequences and was 603 bp in length. Four sequences were retrieved from GenBank and were included in the analyses (Table 5).

Bayesian Inference (BI) phylogenetic analysis was performed on the concatenated dataset using MrBayes v. 3.2.7 (Ronquist et al. 2012). The dataset was partitioned according to loci, and the substitution models used were those suggested by PartitionFinder (Lanfear et al. 2016). Two independent analyses (nruns = 2) were performed simultaneously for 2*10^6^ generations. Parameter estimates were summarised after excluding the first 25% as burn-in. The 50% majority rule consensus tree was generated from the two simultaneous analyses and was visualised using FigTree v.1.4.4. A burn-in value of 25% was set during consensus tree generation. *Alpiscorpiusgermanus* (Koch, 1837) and *Tetratrichobothriusflavicaudis* (DeGeer, 1778) were used as outgroup taxa. The phylogenetic tree is presented in Fig. 31.

## ﻿Taxonomy

**Family Euscorpiidae Laurie, 1896**

**Genus *Euscorpius* Thorell, 1876**

### Subgenus Euscorpius Thorell, 1876

#### ﻿ Euscorpius concinnus

Taxon classificationAnimaliaScorpionesEuscorpiidae

(C.L. Koch, 1837)

C7AA013C-C0FD-5995-9997-5588C83C0B4E

**Figure 1.**
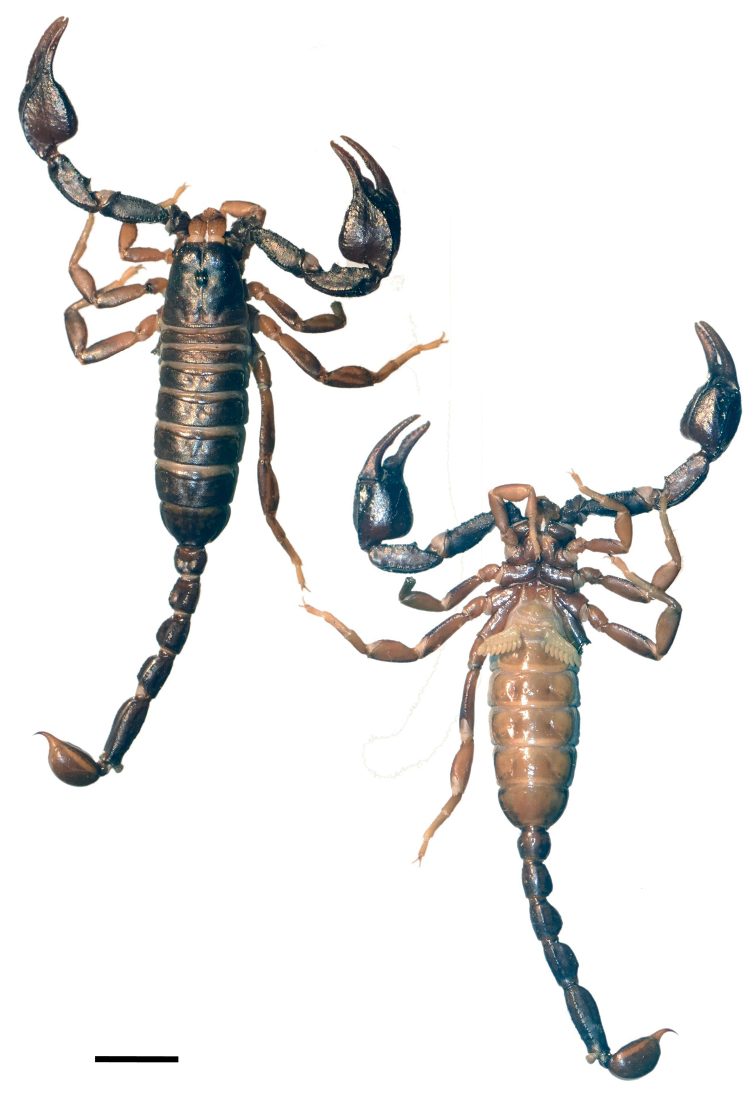
Dorsal and ventral view of *Euscorpiusconcinnus* male topotype. Scale bar: 4.00 mm.

**Figure 2.**
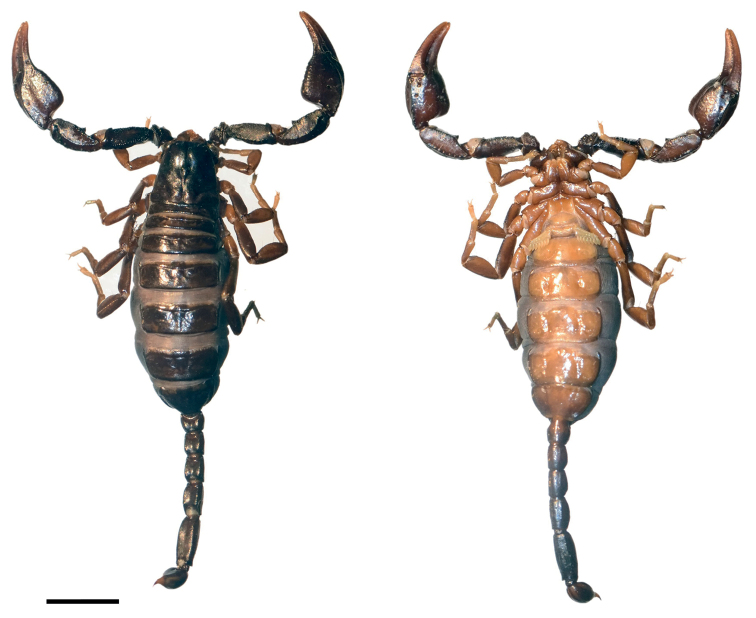
Dorsal and ventral view of *Euscorpiusconcinnus* female topotype. Scale bar: 5.00 mm.

**Figure 3.**
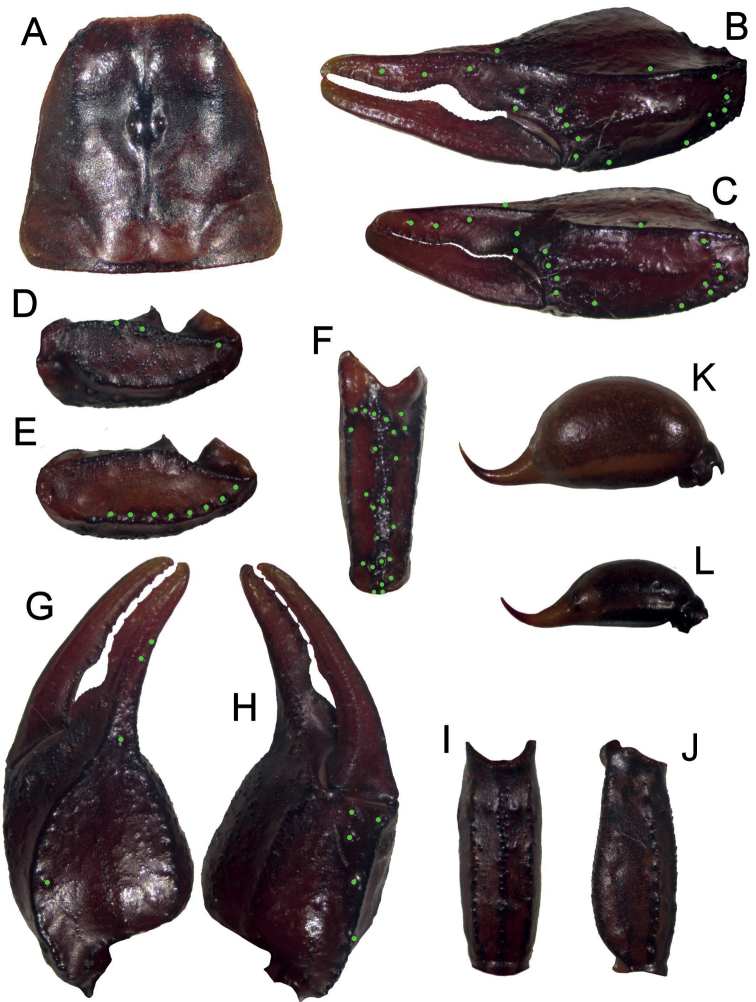
*Euscorpiusconcinnus* topotype **A** carapace **B** external view of chela of adult male **C** external view of chela of adult female **D** dorsal view of pedipalp patella **E** ventral view of pedipalp patella **F** external view of pedipalp patella **G** dorsal view of chela **H** ventral view of chela **I** ventral view of metasomal segment V **J** lateral view of metasomal segment V **K** telson of adult male **L** telson of adult female.

**Figure 4.**
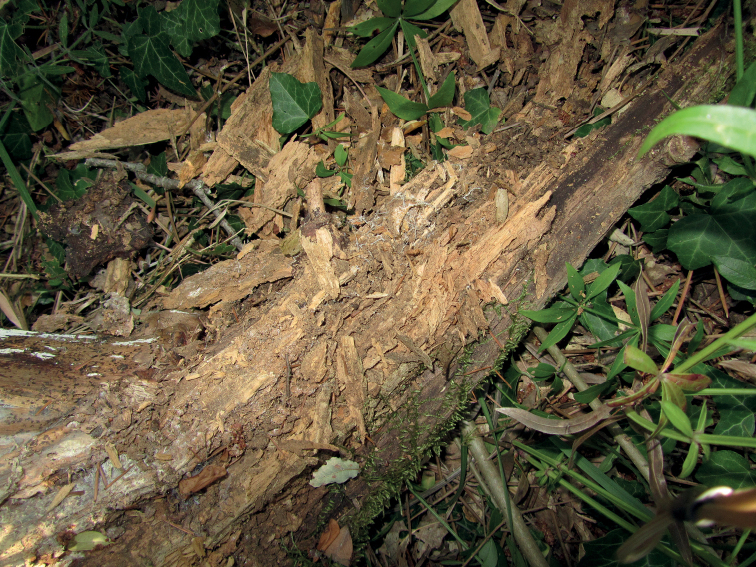
Barked trunk in which some specimens of *Euscorpiusconcinnus* were found.

**Figure 5.**
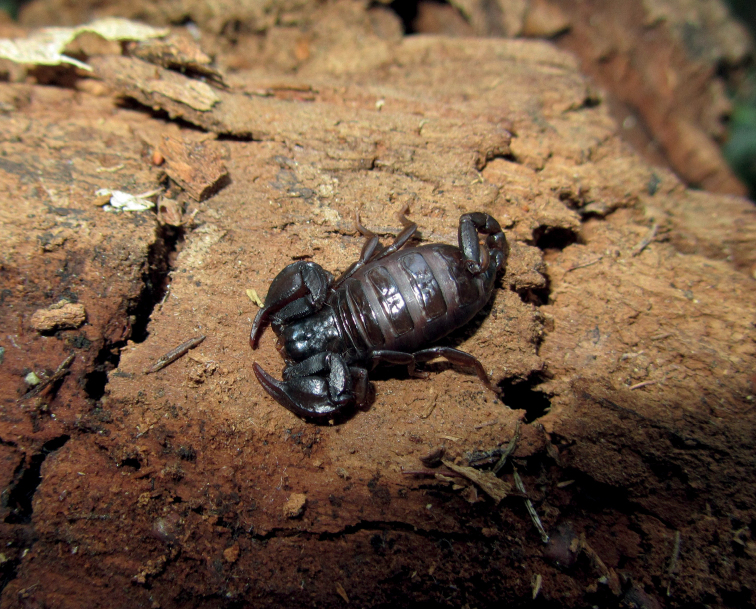
Pregnant *Euscorpiusconcinnus* found under the bark of a trunk.

Figs 1 , 2 , 3 , 4 , 5 , Tables 1 , 3 , 4

##### Type material.

***Holotype***: ♀ (lost), type locality unknown.

***Neotype***: ♀ (VVZC Eut516), Italy, Tuscany, Castelnuovo Berardenga (SI), Ponte a Bozzone, 43.3503333°N, 11.38613889°E, under tree bark, pine wood, 13 October 2003 (V. Vignoli and F. Cicconardi coll.), by designation of Vignoli et al. (2005).

##### Other examined specimens.

**Italy: Tuscany**: near SP408, W of San Giovanni A Cerreto, (SI), 43.350367°N, 11.385845°E, 293, under tree bark, pine wood, 12 August 2013, leg. G. Tropea, S. Tropea, 2 ♂♂, 4 ♀♀ topotypes (GTC 352–358); Apuan Alps, 44.05079°N, 10.26472°E, 810 m, 19 July 2015, leg. G. Tropea, S. Tropea, 2 ♀♀ (GTC 648, 649); Elba Island, lrgs. A. Valle, Bianchi, 4 January 1963, 1 ♂ (MSNB 1593); Elba Island, A. Valle, Bianchi, 8 January 1963, 1 ♂♂ (MSNB 1604); Elba Island, Mt. Perone, pinewood, legs. E. Dominici, 1 ♂ (GTC 462); Valle Benedetta, hills of Livorno, Livorno, 13 May 2012, leg. G. Tropea, 1 ♀ (GTC161–168); Massa, 17 October 2011, leg. A. Rossi, 1 ♂, 1 ♀ (GTC 109, 110); Mt. Albano, Firenze, 1969, leg. Giuliani, 1 ♀♀ (MZUF 9552); Mt. Albano, Firenze, 20 September 1975, leg. Valle, Moretti, 1 ♀ (MSNB 10158); Mt. Corchia, 44.02416°N, 10.27786°E, 708 m, 19 July 2015, leg. G. Tropea, S. Tropea, 2 ♂♂, 3 ♀♀ (GTC 643–647); same data but 44.03448°N, 10.27165°E, 1126 m, 1 ♂, 1 ♀ (GTC 641, 642); Piazza al Serchio (LU), 20 September 1975, leg. A. Valle, G.L. Moretti, 4 ♂♂, 5 ♀♀ (MSNB 10137–10140, 10143, 10147, 10153, 10156, 10157); Resceto (MS), 551–614 m, 11 August 2013, leg. G. Tropea, S. Tropea, 9 ♂♂, 6 ♀♀ (GTC 328–338); near Barberino di Mugello, 44.02430°N, 11.16499°E – 44.02357°N, 11.16451°E, 450–484 m, 12 August 2013, leg. G. Tropea, S. Tropea, 4 ♂♂, 3 ♀♀ (GTC 345–351); near Livorno, 43.570116 N, 10.369947 E, July 2021, leg. Giuliano Tropea, 1 ♀ (GTC). **Emilia Romagna**: Pievepelago (MO), Lago Santo, 22 July 1978, leg. Daccordi, 1 ♀ (MSNV 154/11774); Ponte Modino, Pievepelago, (MO), 44.18564°N, 10.61523°E, 938 m, 20 July 2015, leg. G. Tropea, S. Tropea, 2 ♂♂, 2 ♀♀ (GTC 650–653).

**Table 2.** Measurements (mm) of *Euscorpiusstefaniae* sp. nov. and *E.trejaensis* sp. nov.

		*E.stefaniae* sp. nov.		*E.trejaensis* sp. nov.	
		Holotype ♂	Paratype ♀	Holotype ♂	Paratype ♀
**Total**	**Length**	26.55	29.90	24.71	28.17
**Carapace**	**Length**	4.02	4.68	3.66	4.08
	**Post. width**	4.32	4.92	3.80	4.44
**Metasoma**	**Length**	10.59	11.24	9.75	10.35
**Segment I**	**Length**	1.38	1.49	1.30	1.32
	**Width**	1.62	1.85	1.40	1.57
**Segment II**	**Length**	1.59	1.74	1.40	1.59
	**Width**	1.48	1.62	1.30	1.42
**Segment III**	**Length**	1.80	1.92	1.70	1.80
	**Width**	1.44	1.50	1.23	1.36
**Segment IV**	**Length**	2.16	2.28	2.05	2.16
	**Width**	1.38	1.44	1.20	1.26
**Segment V**	**Length**	3.66	3.81	3.30	3.48
	**Width**	1.38	1.44	1.22	1.26
**Telson**	**Length**	4.08	3.90	3.80	3.30
**Vesicle**	**Length**	2.88	2.58	2.80	2.28
	**Width**	1.56	1.49	1.50	1.29
	**Height**	1.63	1.32	1.50	1.13
**Aculeus**	**Length**	1.20	1.32	1.00	1.02
**Femur**	**Length**	3.30	3.78	3.00	3.30
	**Width**	1.32	1.56	1.16	1.35
**Patella**	**Length**	3.38	3.90	3.18	3.49
	**Width**	1.38	1.74	1.40	1.50
**Chela**	**Length**	6.72	8.13	6.31	7.32
	**Width–A**	2.58	3.03	2.50	2.70
**Movable finger**	**Length**	3.72	4.95	3.80	4.08

##### Known geographic range.

Italy: Tuscany, Emilia Romagna, Marche?, Umbria?, Piemonte?, Liguria (Fig. 32).

##### Diagnosis.

Medium *Euscorpius* species, total length 26–35 mm. Variable colour in adults, from dark brown to blackish, with darker marbling on most of the body, including chelicerae. The number of trichobothria on the pedipalp manus ventral surface is four (V_1–3_ + Et_1_). Trichobothria est and dsb on fixed finger are respectively located distally and proximally to the notch of the fixed finger. The number of trichobothria on the pedipalp patella ventral surface is usually eight or nine (~ 65% and 27%, respectively). The number of trichobothria on pedipalp patella external surface usually is: eb = 4, eb_a_ = 4, esb = 2, em = 4, est = 4, et = 6. Trichobothrium i of the femur is slightly proximal to or at the same level of d. The pectinal teeth number in males is usually eight and in females mostly seven. Dorsal patellar spur well developed. Femur approximately as long as patella but it tends to be slightly shorter than patella. Carapace approximately as long as wide, but it tends to be slightly wider than long. Carinae V_1_ follows an external direction to the trichobothria Et_1_, without forming a Y-shape. Spinules on legs ending with a Y-shape. Ventrolateral and ventromedian carina on metasomal segment V well formed by small spaced serrulated granules.

##### Trichobothrial and pectinal teeth count variation.

The variation observed in 59 examined specimens (28 ♂♂, 31 ♀♀) is given below.

Pectinal teeth in males (*n* = 56): 7/7 (1), 8/7 (1), 8/8 (16), 8/9 (6), 9/9 (2), 9/10 (1), 10/10 (1); in total, 7 in 5.36% (3), 8 in 69.64% (39), 9 in 19.64% (11), and 10 in 5.36% (3); mean = 8.25, SD = 0.64.

Pectinal teeth in females (*n* = 62): ?/? (1), 6/6 (1), 6/7 (4), 7/7 (17), 7/8 (3), 8/? (1), 8/8 (4); in total, 6 in 10.17% (6), 7 in 69.49% (41) and 8 in 20.34% (12); mean = 7.10, SD = 0.55.

Pedipalp patella trichobothria Pv (*n* = 118): 7/7 (1), 8/7 (6), 7/9 (1), 8/8 (32), 8/9 (7), 9/9 (12); in total, 7 in 7.63% (9), 8 in 65.25% (77) and 9 in 27.12% (32); mean = 8.19, SD = 0.56.

Pedipalp patella trichobothria Pe (*n* = 118): et = 4/6 (1), 5/6 (7), 6/6 (43), 6/7 (5), 7/7 (3); in total, 5 in 5.93% (7), 6 in 83.90% (99), and 7 in 9.32% (11); mean = 6.03, SD = 0.39;

est = 4/3 (1), 4/4 (56), 4/5 (1), 5/5 (1); em = 4/3 (3), 4/4 (56); esb = 1/2 (1), 2/2 (58); eb_a_ = 3/4 (2), 4/4 (57); eb = ¾ (3), 4/4 (56).

##### Description of the hemispermatophore.

Type A. It has a well-developed lamina tapered distally; well-developed basal constriction present; truncal flexure present; median projection with lde, ldi, and lb; internal projection distally with 8–10 tines in its crown. The number and the shape of tines of the crown varied between specimens and between the right and the left hemispermatophores.

##### Comments.

*Euscorpiusconcinnus* was originally described using very limited characters, not very useful in identifying the species. The main characters given by Koch (1837) were Pv = 8 and dark red-brown colouring, both can be shared by different species of Euscorpiinae. The location where the specimen was collected is unknown, therefore relying on this data is not possible too. It would probably have been more appropriate to consider this species a nomen dubium, but Vignoli et al. (2005), perhaps influenced by Di Caporiacco (1950), established a neotype for it from Siena (Tuscany, Italy). This species, like the others treated here, can be considered a cryptic species, difficult to identify without data regarding the exact geographical area of collection or DNA analysis. According to our data, this species is basal to the other species treated in the phylogeny based on concatenated 16S rDNA and COI markers (Fig. 31). Probably the present populations are the result of expansions, extinctions, bottleneck, and recolonisations, taking place several times due to climatic changes during the Pleistocene. The sequence divergence in 16S marker between *E.concinnus* and the remaining species of the *E.concinnus* group ranges from 2.7% to 3.4%, the latter value being higher than that between *E.concinnus* and *E.tergestinus*, which is 3.1%. As for the trichobothrial Pv number, *E.concinnus* shows an average of 8.19, clearly lower than that of *E.niciensis* stat. nov., but higher than of the other three species treated herein. The highest percentage was of Pv = 8 in 65.25%, i.e., similar to that of *E.latinus* sp. nov. and *E.trejaensis* sp. nov., but significantly lower than *E.stefaniae* sp. nov. with 81.67%, and clearly higher than *E.niciensis* stat. nov. with 25%; and a percentage of Pv = 9 in the 27.12%, which is clearly higher than that of all species except *E.niciensis* stat. nov. which has a percentage of Pv = 9 of 68.89%. It must be stated that this higher value in *E.concinnus* could be brought about by its heterogeneity in its distributional area. In fact, in some areas this species may have a tendency to higher or lower values, and also to morphological differences, which could suggest further taxonomic divisions in it, as well as for *E.niciensis* stat. nov. (GT, in progress).

**Table 3.** Percentage of the number of the pectinal teeth found in the examined specimens. Abbreviations: *conc. = E.concinnus*; *lati. = E.latinus* sp. nov.; *nici. = E.niciensis* stat. nov.; *stef. = E.stefaniae* sp. nov.; *trej. = E.trejaensis* sp. nov.

Dp count	Dp ♂ %					Dp ♀ %				
	*conc.*	*lati.*	*nici.*	*stef.*	*trej.*	*conc.*	*lati.*	*nici.*	*stef.*	*trej.*
**6**	–	–	–	–	–	10.17	17.91	4.17	-	21.17
**7**	5.36	6.90	1.28	–	8.14	69.49	65.67	61.46	75	71.53
**8**	69.64	77.59	32.05	–	80.23	20.34	14.92	33.33	25	6.57
**9**	19.64	17.24	60.26	92.86	10.46	–	–	1.04	–	0.73
**10**	5.36	-	2.56	7.14	1.16	–	–	–	–	–
**Average**	**8.25**	**8.10**	**8.72**	**9.07**	**8.05**	**7.10**	**6.94**	**7.31**	**7.25**	**6.87**

Ythier (2011) assigned some specimens from France to *E.concinnus* and *E.tergestinus* and reports a Pv range of seven and eight for *E.concinnus* and from seven to eleven (nine) for *E.tergestinus*. However, no additional data are provided for these claims. *Euscorpiustergestinus* is limited to the Balkans and to the Italian areas near the border with Slovenia (Tropea, 2013). Considering Ythier’s data, his *E.tergestinus* and *E.concinnus* populations probably were *E.niciensis* stat. nov. We found no populations with such low fixed trichobothrial data neither in the specimens examined from France nor in those from western Liguria in Italy. Therefore, in the absence of further data, we consider that the range of this species is limited within Italy.

**Table 4.** Percentage of the number of the trichobothrial series Pv and Pe-et found in the examined specimens. Abbreviations: T count = number of the trichobothria; *conc. = E.concinnus*; *lati. = E.latinus* sp. nov.; *nici. = E.niciensis* stat. nov.; *stef. = E.stefaniae* sp. nov.; *trej. = E.trejaensis* sp. nov.

T count	Pv %					Pe–et %				
	*conc.*	*lati.*	*nici.*	*stef.*	*trej.*	*conc.*	*lati.*	*nici.*	*stef.*	*trej.*
**5**	–	–	–	–	–	5.93	17.28	6.67	10.00	10.69
**6**	–	–	–	–	2.65	83.90	79.01	81.67	83.33	83.02
**7**	7.63	39.68	2.78	13.33	34.07	9.32	1.23	11.67	5.00	4.40
**8**	65.25	60.32	25.00	81.67	61.95	–	–	–	–	–
**9**	27.12	–	68.89	3.33	1.33	–	–	–	–	–
**10**	–	–	3.33	–	–	–	–	–	–	–
**Average**	**8.19**	**7.60**	**8.73**	**7.90**	**7.62**	**6.03**	**5.79**	**6.05**	**5.95**	**5.90**

The current distributional range of *E.concinnus* is not easily delineable yet. We consider it an endemic species of Italy, found in Tuscany, Liguria, and Emilia Romagna, and with doubtful presence in the regions of Piedmont, Lombardy, Umbria and Marche. *Euscorpiusconcinnus* was found up to an altitude of 1126 m a.s.l., in the Apuan Alps. It is found mostly in woods, both under stones and bark, but also in humid microhabitats in pine forests, and more rarely in human-made constructions, in wall cracks, and in the areas around the walls.

#### ﻿ Euscorpius latinus sp. nov.

Taxon classificationAnimaliaScorpionesEuscorpiidae

3BE61A0C-D2EC-551D-8780-BB061C6E51D7

http://zoobank.org/14CD5A7C-378B-4C3E-8664-C9EA5CD0CB1D

**Figure 6.**
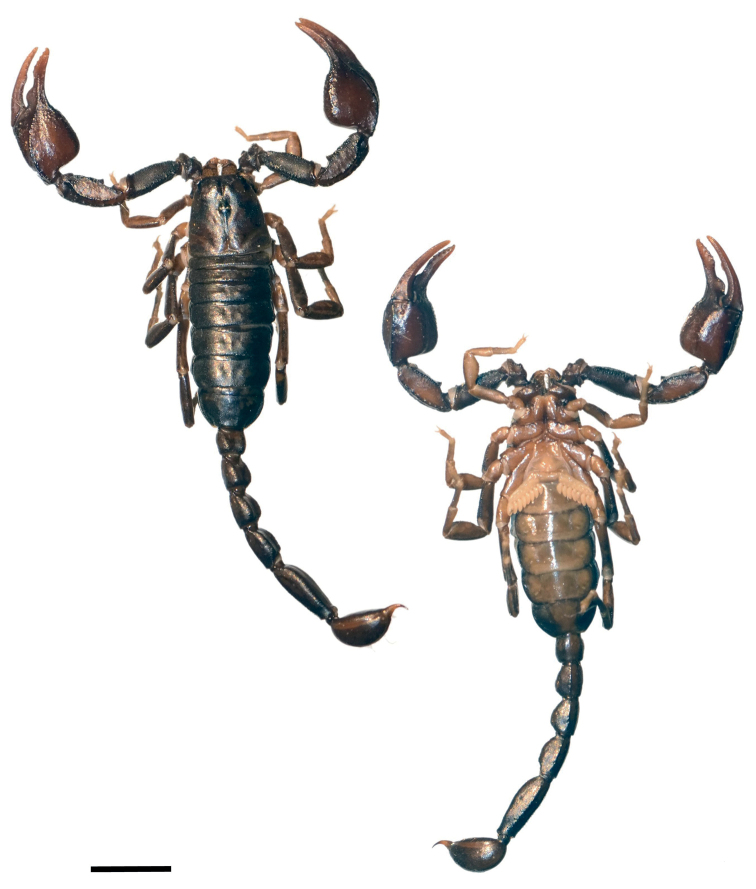
Dorsal and ventral views of *Euscorpiuslatinus* sp. nov. male holotype. Scale bar: 4.20 mm.

**Figure 7.**
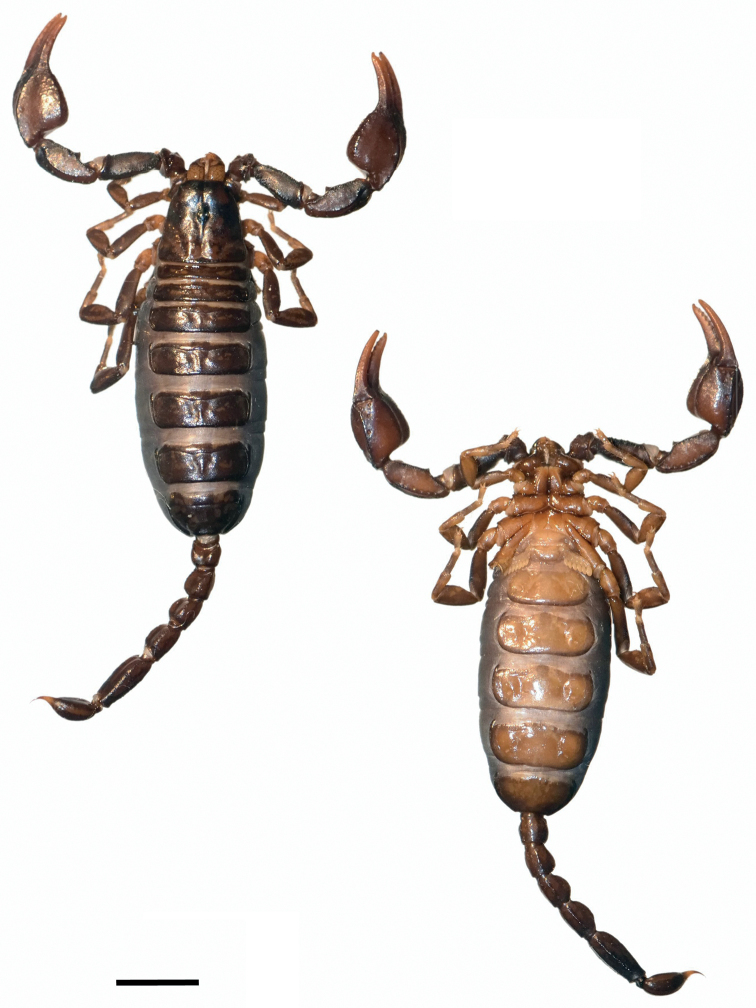
Dorsal and ventral view of *Euscorpiuslatinus* sp. nov. female paratype. Scale bar: 4.20 mm.

**Figure 8.**
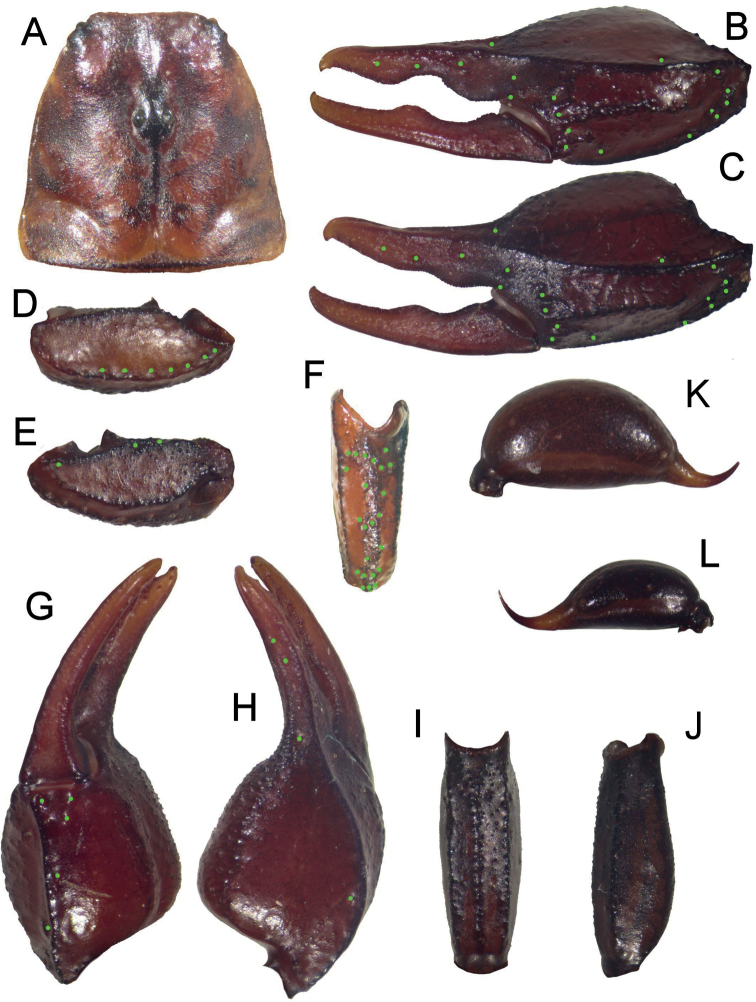
*Euscorpiuslatinus* sp. nov. male holotype except **C** and **N**, that are of a female paratype **A** carapace **B** external view of chela of adult male **C** external view of chela of adult female **D** ventral view of pedipalp patella **E** dorsal view of pedipalp patella **F** external view of pedipalp patella **G** ventral view of chela **H** dorsal view of chela **I** ventral view of metasomal segment V **J** lateral view of metasomal segment V **K** telson of adult male **L** telson of adult female.

**Figure 9.**
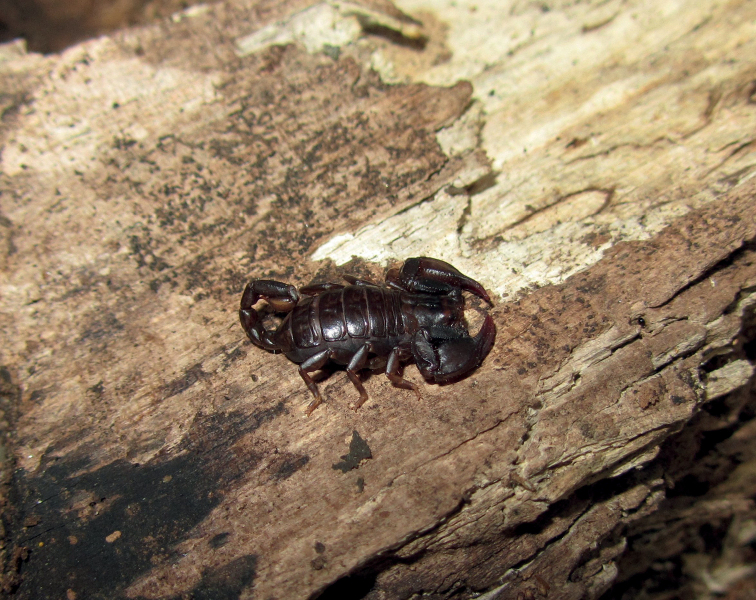
Pregnant *Euscorpiuslatinus* sp. nov. found under the bark of a trunk.

**Figure 10.**
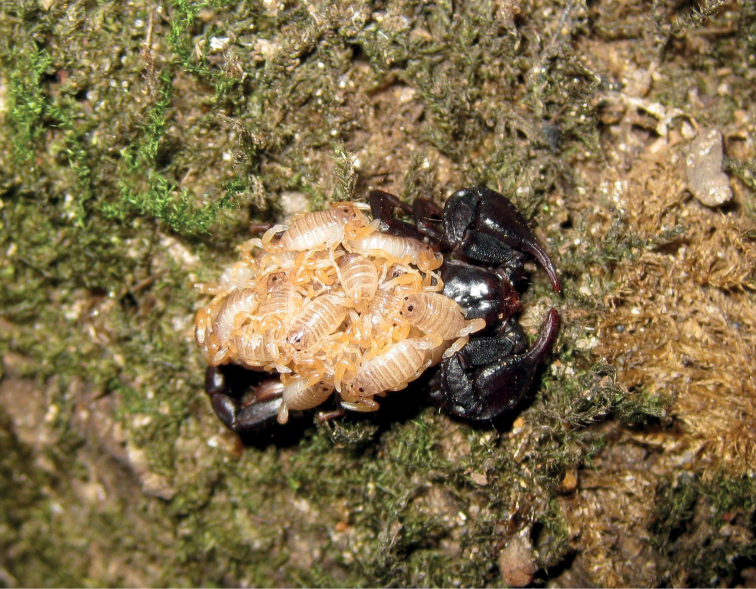
*Euscorpiuslatinus* sp. nov. photographed in its habitat with first instar litter on its back.

**Figure 11.**
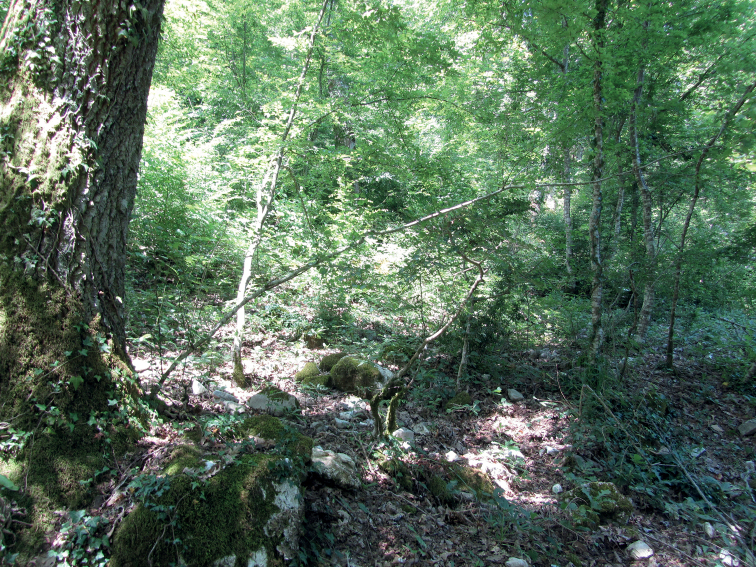
Example of the habitat of *Euscorpiuslatinus* sp. nov.

**Figure 12.**
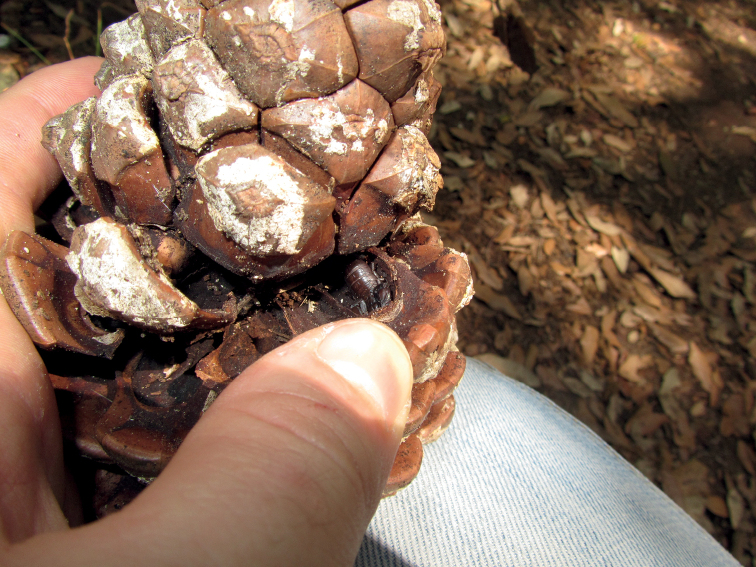
Pregnant specimen of *Euscorpiuslatinus* sp. nov. found inside a pine cone.

Figs 6 , 7 , 8 , 9 , 10 , 11 , 12 , Tables 1 , 3 , 4

##### Type material.

***Holotype***: ♂, Italy, Latium, Lepini Mts, near Montelanico (RM), 470 m a.s.l., 41.631314°N, 13.026798°E, 20 June 2013, leg. G. Tropea (GTC).

***Paratypes*: Italy: Latium**: Lepini Mts, near Montelanico (RM), 470 m a.s.l., 41.631314°N, 13.026798°E, 20 June 2013, leg. G. Tropea, 2 ♂♂, 4 ♀♀ (GTC paratypes); same data but 444 m a.s.l., 41.63219°N, 13.02634°E, 1 ♂, 5 ♀♀ (GTC paratypes); same data but 456–467 m, 41.63118°N, 13.02580°E, 41.63092°N, 13.02530°E, 41.63156°N, 13.02547°E, 12 August 2020, leg G. Tropea, 4 ♂♂, 3 ♀♀ (GTC paratypes); Castel Fusano, Rome, 8 April 2012, leg. G. Tropea, 6 ♂♂, 7 ♀♀ (GTC paratypes); Castel Fusano, Rome, ~ 9 m a.s.l., around to 41,73064°N, 12,31516°E, 22 June 2014, leg. G. Tropea, 3 ♂♂, 4 ♀♀ (GTC paratypes); Near Sabaudia (LT), 18 August 2009, leg. G. Tropea, 3 ♂♂ (GTC paratypes); same data but 5 May 2012, leg. G. Tropea, 3 ♀♀ (GTC paratypes); surroundings of Anticoli Corrado (RM), under stones, 42.012665°N, 12.970851°E, May 2014, leg. A. Massimiani, 2 ♀♀ (GTC paratypes); Near Monterotondo (RM), 109 m a.s.l., 42.06871°N, 12.64305°E, 18 April 2014, leg. G. Tropea, 2 ♂♂, 1 ♀ (GTC paratypes); Simbruini Mts., near Trevi nel Lazio (FR), 1 August 1976, leg. R. Argano, 1 ♂, 1 ♀ (GTC paratypes). **Molise**: Near SP Carovillense, Villa San Michele (IS), 41.74463°N, 14.23146°E, 14 July 2012, leg. G. Tropea, S. Tropea, 5 ♂♂, 1 ♀ (GTC paratypes).

##### Other examined specimens

**(not included in type series). Italy: Latium**: Mt. Gennaro, Lucretili Mts., (RM), ~ 1000 m a.s.l., 24 August 2009, G. Tropea, 2 ♂♂, 2 ♀♀ (GTC).

##### Etymology.

The specific epithet means Latin, due to its range which includes the first area in which the Latins and the Latin language spread, namely the Latium vetus.

##### Known geographic range.

Italy: Latium (left of the Tiber River; Fig. 32).

##### Diagnosis.

A medium-small, *Euscorpius* species, total length 25–34 mm. Colour of adults mostly dark brown with darker marbling on most of the body, including chelicerae, but with rare blackish or medium brown specimens. The number of trichobothria on the pedipalp manus ventral surface is four (*V*_1–3_ + *Et*_1_). Trichobothria est and dsb on fixed finger are respectively located distally and proximally to the notch of the fixed finger. The number of trichobothria on the pedipalp patella ventral surface is usually eight and seven (seven in 39.68% of the pedipalps examined). The number of trichobothria on pedipalp patella external surface is usually: eb = 4, eb_a_ = 4, esb = 2, em = 4, est = 4, et = 6 (5–7). Trichobothrium i of the femur is slightly proximal to or at the same level of d. The pectinal teeth number in males usually is eight (7–9) and in females usually is seven (6–8). Dorsal spur well developed. Femur is slightly shorter than the patella. Carapace tends to be shorter than long. Carinae V_1_ follows an external direction to the trichobothria Et_1_, without forming a Y-shape. Spinules on legs ending with a Y-shape. Ventrolateral and ventromedian carina on metasomal segment V well formed by small, spaced and slightly serrulated granules.

##### Trichobothrial and pectinal teeth count variation.

The variation observed in 63 examined specimens (29 ♂♂ and 34 ♀♀) is given below (left/right asymmetry not specified).

Pectinal teeth in males (*n* = 58): 7/7 (1), 7/8 (2); 8/8 (20), 8/9 (2), 9/9 (4); in total, 7 in 6.90% (4), 8 in 77.59% (45), and 9 in 17.24% (10); mean = 8.10, SD = 0.48.

Pectinal teeth in females (*n* = 67): 5/6 (1), 6/6 (2), 6/7 (7), ?/7 (1), 7/7 (15), 7/8 (6), 8/8 (2); in total, 6 in 17.91% (12), 7 in 65.67% (44), and 8 in 14.92% (10); mean = 6.94, SD = 0.62.

Pedipalp patella trichobothria *Pv* (*n* = 126): 7/7 (15), 7/8 (20), 8/8 (28); in total, 7 in 39.68% (50), and 8 in 60.32% (76); mean = 7.60, SD = 0.49.

Pedipalp patella trichobothria *Pe* (*n* = 81): *et* = 5/4 (1), 5/5 (3), 5/6 (5), 6/1 (1), 6/6 (29), 4/7 (1); in total, 4 in 2.46% (2), 5 in 17.28% (14), 6 in 79.01% (64), and 7 in 1.23% (1); mean = 5.79, SD = 0.49;

est = 4/3 (1), 4/4 (38), 4/5 (2); em = 3/4 (7), 4/4 (33), 4/5 (1); esb = 2/2 (41); eb_a_ = 4/4 (41); eb = 4/4 (39), 4/5 (2).

##### Description of the male holotype.

***Colouration*.** A general dark brown base colour with more or less marked lighter marbling or reticulation, reddish brown, in the less granulated areas, especially of the metasoma, legs, pedipalps and chelicerae; telson mostly dark brown with two ventrally longitudinal pale brown stripes and one for each side, with reddish brown distal part of the sting; pale brown chelicerae with dark brown reticulation; chelae with fingers ranging from pale yellowish brown to dark reddish brown with dark blackish brown reticulation; legs with most ivory tarsus, the basitarsus and tibia are mostly pale brown, but with dark blackish brown marbling externally, almost pale brown internally, the patella and femur are mostly dark with paler spot externally, and mostly pale brown with dark reticulation internally; pectines and genital operculum whitish ivory; sternites are mostly very pale brownish but the most distal is laterally dark blackish brown with the central part pale brown.

***Carapace*.** Almost completely covered by dense fine granules, especially on the dark marbling. The granules in the lateral anterior part are larger; anterior edge is straight with some granules; deep posterior lateral furrows; two pairs of lateral eyes, and a pair of median eyes; length from centre of median eyes to anterior margin is 40.48% of carapace length.

***Mesosoma*.** The tergites are densely covered with a fine granulation; sternites glossy and finely punctuated; small spiracles inclined to ~ 40° downward towards outside.

***Metasoma*.** Dorsal carinae on segments I–IV with spaced granules; ventrolateral carinae on segment I absent, on segment II and III smooth or obsolete, on segments IV, little marked with some small and spaced granule, with small slightly serrulated granules on segment V; ventromedian carinae absent on segment I–III, little marked smooth or obsolete on segment IV, on segment V it consists of small, slightly serrulated granules, which expands like a fan in the most distal part; dorsal and lateral intercarinal surfaces on segments I–IV are mostly finely granulated, especially on dark marbling, while the ventral surfaces are mostly smooth, the V segment is mostly finely granulated.

***Telson*.** Vesicle with a few small granules, with ventral setae of different size, especially near the vesicle/aculeus juncture.

***Pectines*.** Teeth number 8/8; middle lamellae number 5–5; several microsetae on proximal area of teeth, marginal lamellae, and middle lamellae.

***Genital operculum*.** The genital operculum is formed by two longitudinally devised subtriangular sclerites with genital papillae protruding.

***Sternum*.** Pentagonal shape, type 2; slightly wider than long, with a deep posterior emargination.

***Pedipalps*.** Coxa and trochanter with tuberculated carinae. Femur: dorsal internal and external and ventral internal carinae tuberculated; irregular ventral external carinae formed by tubercles just on 1/3 or 1/4 of femur length; external median carinae formed by lightly serrulated tubercles; anterior median carinae formed by some spaced conical tubercles; intercarinal spaces granulated. Patella: dorsal and ventral internal carinae tuberculated; ventral external carinae crenulated; dorsal external carinae slightly crenulated to rough; intercarinal surfaces finely granulated, especially on the dark reticulations near the internal carinae. Dorsal patellar spur well developed. Chela: chelal carina D_1_ is distinct, strong, dark and smooth with a few tubercles; D_4_ is rounded with a few spaced granules; V_1_ is distinct, strong, dark, from rough to smooth, following an external direction to the trichobothria Et_1_; V_3_ is rounded with scattered granules; external carina granulated; intercarinal tegument granulated; the fixed and movable fingers with medium notch and lobe, respectively.

***Finger dentition*.** In the most distal part is present a DD on the tip; MD is formed by very small denticles closely spaced forming an approximately straight line, discontinued at level of the OD; fixed finger has 5/5 OD and 11/10 ID; movable finger has 7/7 OD and 13/15 ID.

***Trichobothria*.** Chela: trichobothria on the pedipalp manus ventral surface V = 2*/3 (V_1–3_) (*the trichobothrium V3 of the left chela is vestigial) + *Et*_1_ = 1/1; trichobothrium V_4_ situated on the external surface of the chela, near the carina V_1_; trichobothrium ratio of et-est/est-dsb is ~ 0.95 and 0.87. Trichobothrium est is distal to the centre of the notch of the fixed finger and dsb is proximal. Patella: Pv = 8/7; et = 6/6, est = 4/4, em = 4/4, esb = 2/2, eb_a_ = 4/4, eb = 4/4. Femur: trichobothrium d is slightly proximal to i, while trichobothrium e is well distal to both d and i, and situated on dorsal surface on dorsal external carina.

***Legs*.** Two pedal spurs present; no tarsal spur; ventral row of tarsus with a total of 12/10 spinules on leg III, of increasing size from proximal to distal, ending with two spinules to form a Y-shape; three main flanking tarsal setae present. Tubercles present on ventral and dorsal surface of all leg femora.

***Chelicerae*.** Typical of the genus *Euscorpius*.

##### Description of the hemispermatophore.

Type A. It has a well-developed lamina tapered distally; well-developed basal constriction present; truncal flexure present; median projection with lde, ldi, and lb; internal projection distally with 9–11 tines in its crown. The number and the shape of tines of the crown varied between specimens and between the right and the left hemispermatophores.

##### Comments.

*Euscorpiuslatinus* sp. nov. is the southernmost species of the *E.concinnus* group. The geographically closest species of the *E.concinnus* group is *E.trejaensis* sp. nov., which seems to be divided from *Euscorpiuslatinus* sp. nov. by the Tiber River. However, in terms of both phylogeny and of DNA sequence divergences, these two species do not seem to be more closely related compared to the others. In fact, according to the concatenated phylogenetic tree 16S rDNA + COI presented herein (Fig. 31), *E.latinus* sp. nov. is placed between *E.concinnus*, which is more basal to it, and the other species, which are apical to it. Regarding the sequence divergence in 16S marker, between *E.latinus* sp. nov. and *E.trejaensis* sp. nov., it ranges from 2.7% to 3.1%, as with *E.niciensis* stat. nov., from 3% to 2.7% with *E.concinnus* and from 3.8% to 4.2% with *E.stefaniae* sp. nov. Morphologically, like the other species of the *E.concinnus* group and the many cryptic species complex that have been described in recent years, *E.latinus* sp. nov. is difficult to identify without reference to the locality of origin or with a limited number of specimens. As for the trichobothrial and pectines teeth values, *E.latinus* sp. nov., together with *E.trejaensis* sp. nov., has the lowest average of Pv, which is 7.60 and 7.62, respectively, having the highest percentage of Pv = 7, i.e., 39.68%, vs. percentages ranging from 2.78% to 13.33% in *E.concinnus*, *E.niciensis* stat. nov. and *E.stefaniae* sp. nov. and a very similar percentage in *E.trejaensis* sp. nov., 34.07. While the percentage of Pv = 8 is very similar to both *E.concinnus* and *E.trejaensis* sp. nov. (from 60.32%–65.25%), it is very different from that of *E.niciensis* stat. nov. and *E.stefaniae* sp. nov. (25% and 81.67, respectively). Dp in males is also quite similar to *E.concinnus* and *E.trejaensis* sp. nov. and very different from *E.niciensis* stat. nov. and *E.stefaniae* sp. nov. As for Pe-et, *E.latinus* sp. nov. has the largest percentage of et = 5, i.e., 17.28%, vs. percentages ranging from 5.93% to 10.69%.

*Euscorpiuslatinus* sp. nov. is the southernmost species of the *E.concinnus* group and its known distribution includes central southern Lazio, on the left bank of the Tiber River, and north-western Molise. Regarding the latter location, this is the first time that a member of the *E.concinnus* group is reported from this region. This could be due to an accidental introduction, but we cannot dismiss the possibility of this area belonging to the natural distributional range of the species. The Apennines in this area are less elevated and more fragmented, and this may have facilitated the colonisation of that region. It remains to be seen if this species continues its distribution to the left of the Tiber River until it reaches Umbria, or if the latter region is inhabited by another species of the *E concinnus* group, as well as whether the species is also present in Campania.

*Euscorpiuslatinus* sp. nov. was found from almost the sea level (e.g., in Castel Fusano, near Ostia (RM)), up to ~ 900 m a.s.l. on the Lucretili Mountains. It was always found in woodlands, mostly mesophilic, but also hygrophilous. It is evident that the species prefers very humid habitats and microhabitats. In these environments, *E.latinus* sp. nov. behaves as a lapidicolous and corticolous species, since it mainly occurs under stones, but also under branches, trunks, and bark, often rotting, as well as inside pine cones. *Euscorpiuslatinus* sp. nov. has not been found in sympatry with other species of scorpions, but it cannot be excluded that rare encounters may occur with *T.flavicaudis* or *Euscorpiusitalicus* (Herbst, 1800) which prefer more rural and less humid habitats than completely natural and very humid ones as *E.latinus* sp. nov.

#### ﻿ Euscorpius stefaniae sp. nov.

Taxon classificationAnimaliaScorpionesEuscorpiidae

594BB026-DAA8-5E28-B747-321171344696

http://zoobank.org/D8A27F9B-258B-43B7-B27A-0D5F675FFEB1

**Figure 13.**
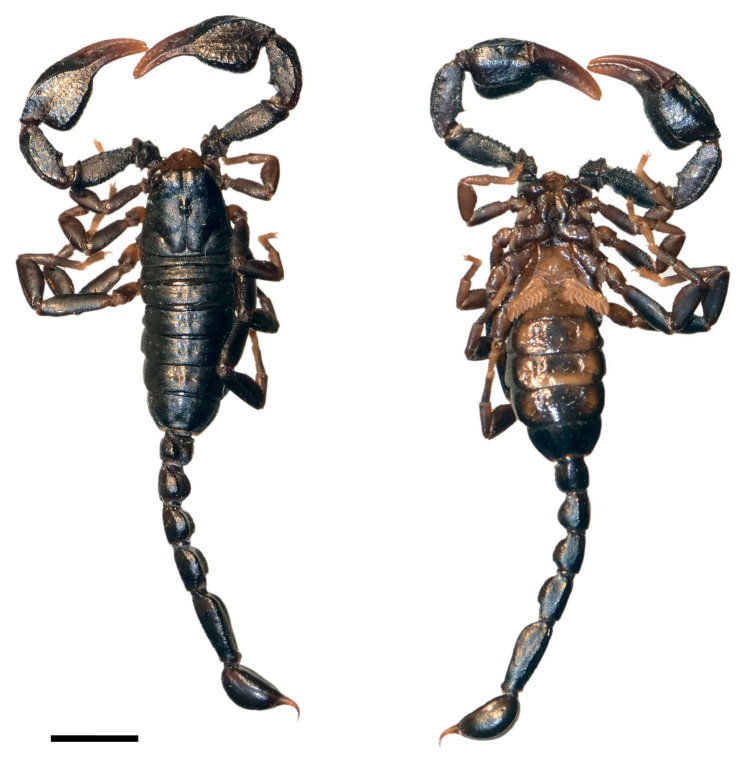
Dorsal and ventral view of *Euscorpiusstefaniae* sp. nov. male holotype. Scale bar: 4.00 mm.

**Figure 14.**
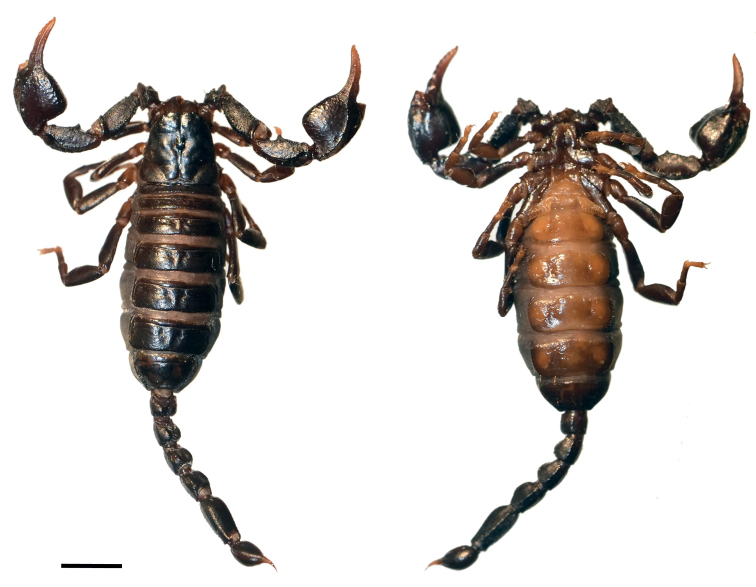
Dorsal and ventral view of *Euscorpiusstefaniae* sp. nov. female paratype. Scale bar: 4.00 mm.

**Figure 15.**
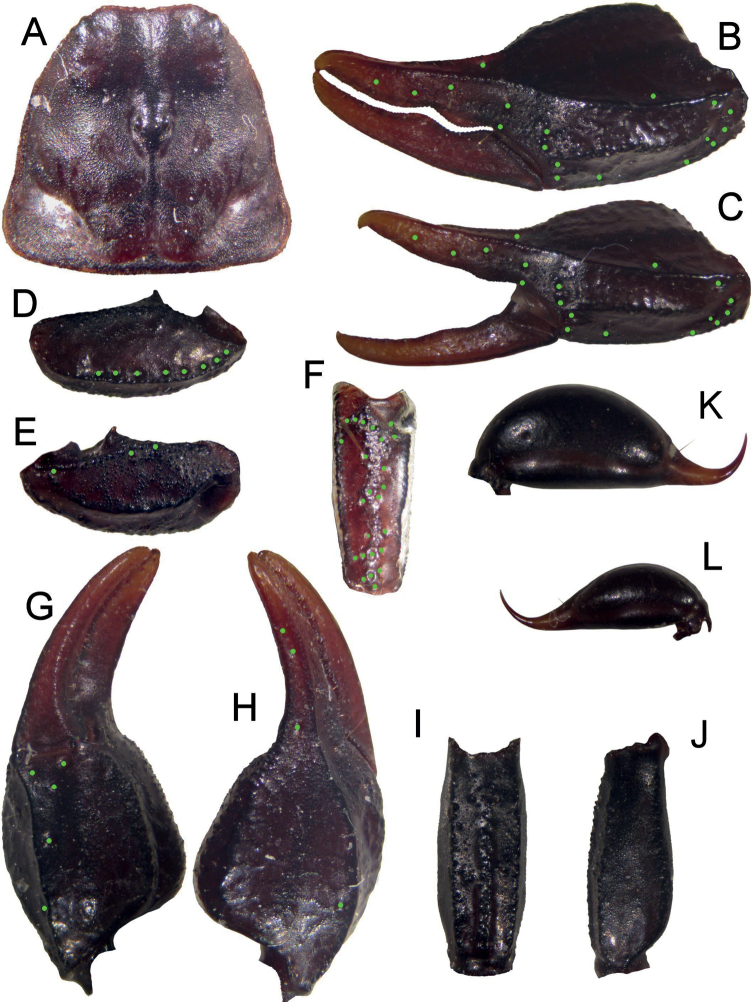
*Euscorpiusstefaniae* sp. nov. male holotype except Figs **C** and **N**, that are of a female paratype **A** carapace **B** external view of chela of adult male **C** external view of chela of adult female **D** ventral view of pedipalp patella **E** dorsal view of pedipalp patella **F** external view of pedipalp patella **G** ventral view of chela **H** dorsal view of chela **I** ventral view of metasomal segment V **J** lateral view of metasomal segment V **K** telson of adult male **L** telson of adult female.

**Figure 16.**
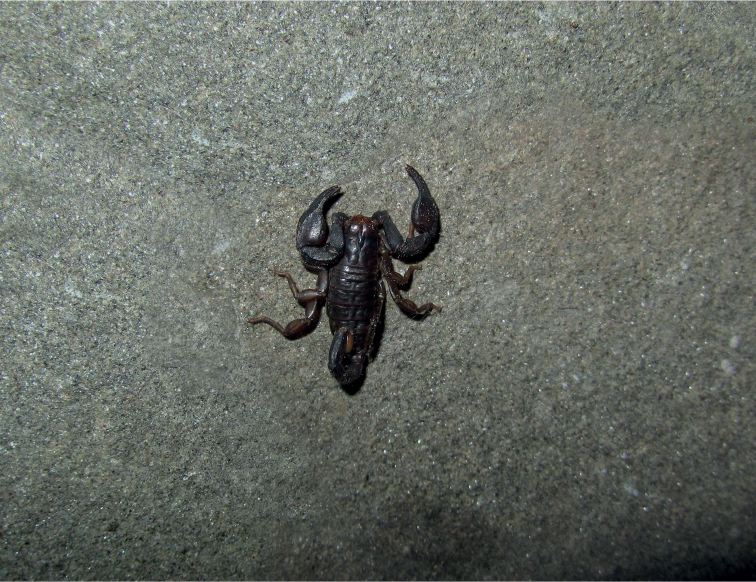
Live specimens of *Euscorpiusstefaniae* sp. nov.

Figs 13 , 14 , 15 , 16 , Table 2 , 3 , 4

##### Type material.

***Holotype***: ♂, Italy, Veneto, Euganean Hills, August 2017, leg. S. Tropea, (GTC).

***Paratypes*: Italy: Veneto**: Euganean Hills, August 2017, leg. S. Tropea, 9 ♂♂, 12 ♀♀ paratypes (GTC); same data but August 2013, leg. S. Tropea, 2 ♂♂ imm., 3 ♀♀ (which 1 imm.) paratypes (GTC).

##### Other examined specimens

**(not included in type series). Italy, Veneto**: Euganean Hills, 18 August 2012, leg. M. Fontana, 2 ♂♂ imm., 1 ♀ imm. (GTC).

##### Etymology.

The specific epithet is in homage to the sister of the first author, Stefania Tropea, for her kind support and enthusiasm shown in several field trips.

##### Known geographic range.

Italy: Veneto (Fig. 32).

##### Diagnosis.

Medium *Euscorpius* species, total length 27–33 mm. The adults are usually blackish coloured, with more or less marked reddish brown marbling, in the less granulated areas, and chelae with fingers ranging from pale yellowish brown to dark reddish brown with blackish reticulation. Some specimens may be reddish brown. The number of trichobothria on the pedipalp manus ventral surface is four (V_1–3_ + Et_1_). Trichobothria est and dsb on fixed finger are respectively located distally and proximally to the notch of the fixed finger. The number of trichobothria on the pedipalp patella ventral surface usually is eight (in 81.67% of the pedipalps examined). The number of trichobothria on pedipalp patella external surface usually is: eb = 4, eb_a_ = 4, esb = 2, em = 4, est = 4, et = 6. The pectinal teeth number in males usually is nine (in 92.86% of the pectines examined) and in females mostly seven (seven or eight). Dorsal patellar spur well developed. Femur usually slightly shorter than patella. Carapace approximately as long as wide, but it can be both slightly longer and shorter. Carinae V_1_ follows an external direction to the trichobothria Et_1_, without forming a Y-shape. Spinules on legs ending with a Y-shape. Ventrolateral and ventromedian carina on metasomal segment V formed by small serrulated granules.

##### Trichobothrial and pectinal teeth count variation.

The variation observed in 30 examined specimens (14 ♂♂ and 16 ♀♀) is given below.

Pectinal teeth in males (*n* = 28): 9/9 (12), 9/10 (1), 10/9 (1); in total, 9 in 92.86% (26/28) and 10 in 7.14% (2/28); mean = 9.07, SD = 0.26.

Pectinal teeth in females (*n* = 32): 7/7 (11), 8/7 (2), 8/8 (3); in total, 7 in 75% (24/32) and 8 in 25% (8/32); mean = 7.25, SD = 0.44.

Pedipalp patella trichobothria Pv (*n* = 60): 7/7 (2), 7/8 (2), 8/6 (1), 8/7 (2), 8/8 (21), 8/9 (1), 9/8 (1); in total, 7 in 13.33% (8/60), 8 in 81.67% (49/60) and 9 in 3.33% (2/60); mean = 7.90, SD = 0.40.

Pedipalp patella trichobothria Pe (*n* = 60): *et* = 5/5 (2), 6/1 (1), 6/5 (2), 6/6 (23), 6/7 (1), 7/7 (1); in total, 5 in 10% (6/60), 6 in 83.33% (50/60) and 7 in 5% (3/60), mean = 5.95, SD = 0.39;

est = 2/4 (1), 3/2 (1), 3/3 (1), 3/4 (1), 2 (4/3), 4/4 (24); em = 3/3 (1), 3/4 (1), 4/3 (1), 4/4 (27); esb = 2/2 (30); eb_a_ = 3/3 (1), 4/3 (1), 4/4 (28); eb = 4/3 (1), 4/4 (27), 4/5 (1), 5/4 (1).

##### Description of the male holotype.

***Colouration*.** A general black base colour with more or less marked paler marbling or reticulation, reddish brown, in the less granulated areas, especially of the metasoma, legs, pedipalps and chelicerae; telson mostly black with two ventrally longitudinal pale brown stripes and one for each side, with yellowish pale brown sting; pale brown chelicerae with dark brown reticulation; chelae with fingers ranging from pale yellowish brown to dark reddish brown with blackish reticulation; legs with almost completely yellowish tarsus, the basitarsus and tibia are especially internally yellowish with dark blackish brown marbling, the patella and femur are mostly dark with paler marbling; pectines and genital operculum yellowish; sternites range from almost completely black to the most distal to yellowish marbled brown of the most proximal.

***Carapace*.** Almost completely covered by a dense granulation; anterior edge is mostly straight and granulate; deep posterior lateral furrows; two pairs of lateral eyes, and a pair of median eyes; length from centre of median eyes to anterior margin is 44% of carapace length.

***Mesosoma*.** The tergites are thickly granulated; sternites glossy and finely punctuated; small spiracles inclined to ~ 45° downward towards outside.

***Metasoma*.** Dorsal carinae on segments I–IV with spaced granules; ventrolateral carinae on segment I absent, on segment II and III smooth or obsolete, on segments IV rouge, on segment V slightly serrulated granules are present; ventromedian carinae absent on segment I–IV, on segment V it consists of small, slightly serrulated granules; intercarinal surfaces are mostly finely granulated with some area, especially those of paler colour, smooth.

***Telson*.** Vesicle mostly smooth, with ventral setae of different size, especially near the vesicle/aculeus juncture.

***Pectines*.** Teeth number 9/9; middle lamellae number 6/5; several microsetae on proximal area of teeth, marginal lamellae, and middle lamellae.

***Genital operculum*.** The genital operculum is formed by two longitudinally devised subtriangular sclerites with genital papillae protruding.

***Sternum*.** Pentagonal shape, type 2; slightly wider than long, with a deep posterior emargination.

***Pedipalps*.** Coxa and trochanter with tuberculated carinae. Femur: dorsal internal and external and ventral internal carinae tuberculated; irregular ventral external carinae formed by tubercles just on 1/3 or 1/4 of femur length; external median carinae formed by lightly serrulated tubercles; anterior median carinae formed by some spaced conical tubercles with three macrosetae; intercarinal spaces granulated. Patella: dorsal and ventral internal carinae tuberculated; ventral external carinae crenulated; dorsal external carinae slightly crenulated to rough; intercarinal surfaces finely granulated, especially on the dark reticulations near the carinae. Dorsal patellar spur well developed. Chela: chelal carina D_1_ is distinct, strong, dark and smooth with a few tubercles proximally; D_4_ is rounded with a few spaced granules; V_1_ is distinct, strong, dark, from rough to smooth, following an external direction to the trichobothria Et_1_; V_3_ is rounded with scattered granules; external carina granulated; intercarinal tegument granulated; the fixed and movable fingers with little marked notch and lobe, respectively.

***Finger dentition*.** In the most distal part is present a DD on the tip; MD is formed by very small denticles closely spaced forming an approximately straight line, discontinued at level of the OD; fixed finger has 6/6 OD and 11/12 ID; movable finger has 8/8 OD and 16/16 ID.

***Trichobothria*.** Chela: trichobothria on the pedipalp manus ventral surface V = 3/3 (V_1–3_) + Et_1_ = 1/1; trichobothrium V_4_ situated on the external surface of the chela carina near the carina V_1_; trichobothrium ratio of et-est/est-dsb is ~ 1. Patella: Pv = 8/8; et = 6/6, est = 4/4, em = 4/4, esb = 2/2, eb_a_ = 4/4, eb = 4/4. Femur: trichobothrium d is slightly proximal to i, while trichobothrium e is well distal to both d and i, and situated on dorsal surface on dorsal external carina.

***Legs*.** Two pedal spurs present; no tarsal spur; ventral row of tarsus with a total of 9/12 spinules on leg III, of increasing size from proximal to distal, ending with a Y-shape; three main flanking tarsal setae present. Tubercles present on ventral and dorsal surface of all leg femora.

***Chelicerae*.** Typical of the genus *Euscorpius*.

##### Description of the hemispermatophore.

Type A. It has a well-developed lamina tapered distally; well-developed basal constriction present; truncal flexure present; median projection with lde, ldi, and lb; internal projection distally with seven or eight tines in its crown. The number and the shape of tines of the crown varied between specimens and between the right and the left hemispermatophores.

##### Comments.

*Euscorpiusstefaniae* sp. nov. is the only species of *E.concinnus* group occurring in northeastern Italy. In fact, to the east of this species’ range, the genus *Euscorpius* s. str. is mostly represented by *E.tergestinus*, the most phylogenetically closely related species to *E.concinnus* group. This might suggest that *E.stefaniae* sp. nov. could be the most basal species of the latter species group, given that the dispersal and speciation of the genus *Euscorpius* s. str. seems to have proceeded in the direction from east to west. The dispersal and speciation probably continued westwards, following the Prealps and then the Apennines toward the south, which were the only possible dispersal routes since the relatively recent Padan Plain did not exist at that time. *Euscorpiusstefaniae* sp. nov. could be a relict species, and the others species of the E.concinnus group speciated and colonised new areas approximately at the same period, but at a later time than *E.stefaniae* sp. nov., after the extinction of the ancestral population from most areas, as suggested by the 16S phylogeny (not shown here). However, in the concatenated tree inferred with 16S + COI markers, the most basal species, placed after *E.tergestinus*, is *E.concinnus*, with *E.stefaniae* sp. nov. in the apical position. This could be explained by the extinction of all ancestral populations in the north, and then the subsequent dispersal and speciation from areas, for example, of Tuscany. *Euscorpiusstefaniae* sp. nov. is well separated from the other species of *E.concinnus* group with a divergence in 16S of 3.1%–4.2%, almost equal to that between *E.tergestinus* and *E.concinnus*. Morphologically, like the remaining species of the *E.concinnus* group and the numerous cryptic species complexes described in recent years, *E.stefaniae* sp. nov. is difficult to distinguish without knowing its origin and having a large sample size. However, it has morphological characters that show a good separation from other related species: *E.stefaniae* sp. nov. has the highest mean Dp in males (9.07) and the highest percentage of Dp = 9 (92.86%). The related species, on average, range from 8.05 to 8.25 except *E.niciensis* stat. nov., which has an average of 8.72, and Dp = 9 ranging from 10.46% to 19.64%, except *E.niciensis* stat. nov. which has 60.26%, a much lower value. Also, the average is higher in females compared to the other *E.concinnus* group species, but not significantly. Another interesting value in this species is Pv = 8 in 81.67%. In fact, although considered a typical value for the *E.concinnus* group, this species has the higher percentage with this number than the other species which have values ranging from 25% to 65%.

The Euganean Hills are a group of hilly reliefs of volcanic origin with a height ranging from ~ 200–600 m, which rises almost isolated to the southwest of Padua. The climate and microclimates on the Euganean Hills can vary greatly depending on the area. They can be mainly divided into sub-Mediterranean, typical of the south-facing slopes, and sub-mountain typical of the north-facing slopes. Depending on the slope and area, the woods can be quite humid. The specimens were found mostly under stones in the less humid areas and mostly under bark in the more humid areas.

#### ﻿ Euscorpius trejaensis sp. nov.

Taxon classificationAnimaliaScorpionesEuscorpiidae

39EE936C-2B90-598F-B53B-9FABD63F123D

http://zoobank.org/314EBC7E-4292-4033-A5A8-F926FDB386E0

Figs 17 , 18 , 19 , 20 , 21 , 22 , 23 , 24 , 25 , 26 , 27 , Table 2 , 3 , 4

##### Type material.

***Holotype***: ♂, Italy, Latium, near Calcata (VT), Treja’s valley, 42.21953°N, 12.4175°E, 21 June 2020, leg. G. Tropea, (GTC 1184).

**Figure 17.**
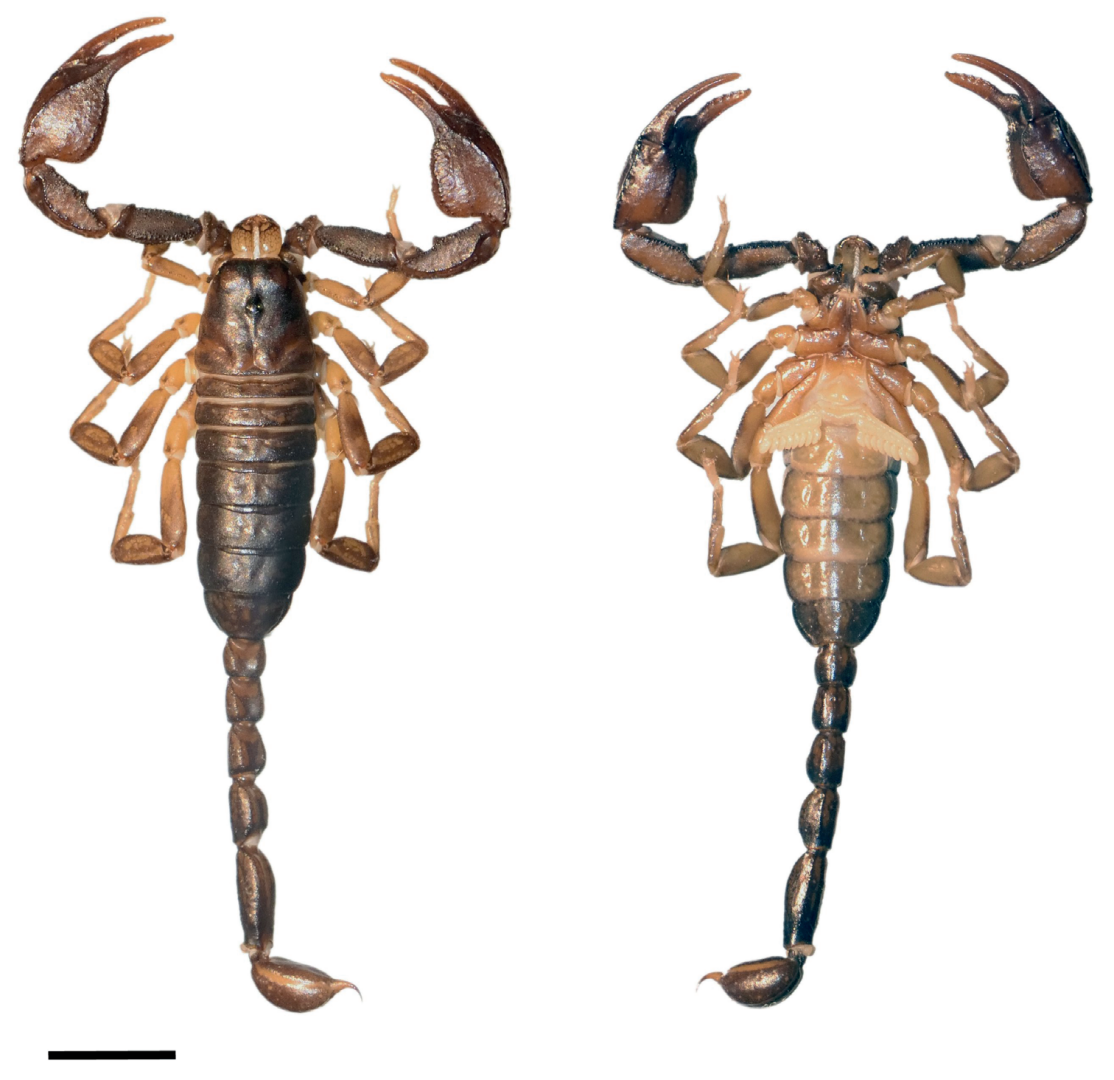
Dorsal and ventral view of *Euscorpiustrejaensis* sp. nov. male holotype. Scale bar: 4.00 mm.

***Paratypes*: Italy: Latium**: near Calcata (VT), Treja’s valley, 42.21953°N, 12.4175°E, 21 June 2020, leg. G. Tropea, 4 ♀♀ (GTC); near Calcata (VT), Treja’s valley, 42.2080833°N, 12.4141667°E, 13 June 2009, leg. G. Tropea, S. Tropea, 3 ♀♀ (GTC); near Calcata (VT), Treja’s valley, 28 July 2009, leg. G. Tropea, 2 ♂♂, 2 ♀♀ (GTC); same data but 2 April 2012, leg. G. Tropea, 3 ♀♀ (GTC); same data but 42.21413°N, 12.41629°E, 114 m, 6 May 2014, leg. G. Tropea, 3 ♂♂, 9 ♀♀ (GTC 498–508, 851); same data but 42.21930°N, 12.418°E – 42.2188889°N, 12.4154°E, between the 100 e i 150 m, 8 April 2018, leg. G. Tropea, 7 ♀♀ (GTC 1092–1098); Rio Fiume, Monti della Tolfa, 112 m, 42.07565°N, 11.96410°E, 11 May 2014, leg. G. Tropea, 5 ♂♂, 12 ♀♀ (GTC 509–525); Rio Fiume, Monti della Tolfa, 9 April 2012, leg. G. Tropea, 1 ♂, 3 ♀♀ (GTC 128–131).

**Figure 18.**
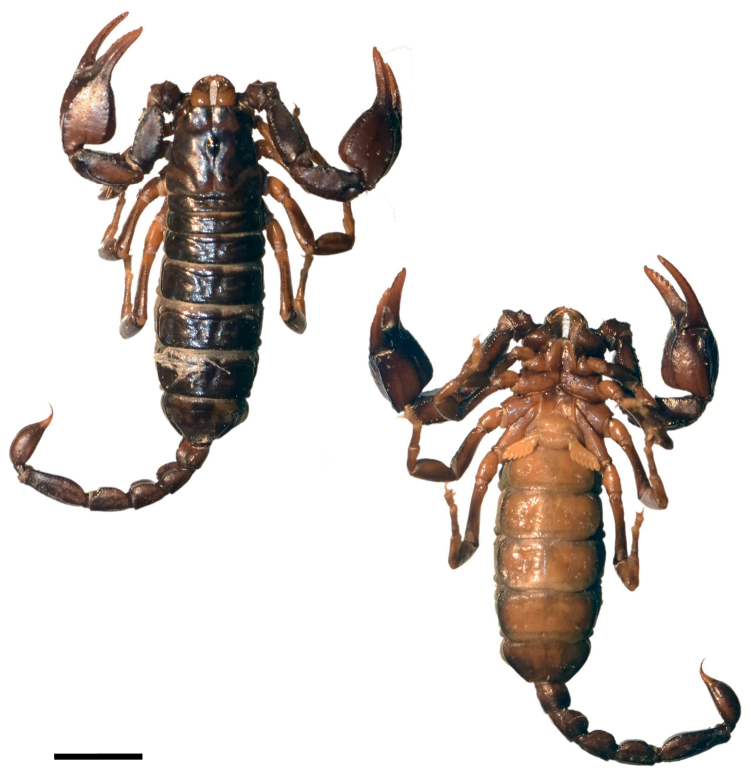
Dorsal and ventral view of *Euscorpiustrejaensis* sp. nov. female paratype. Scale bar: 4.00 mm.

##### Other examined specimens

**(not included in type series). Italy: Latium**: near Calcata (VT), Treja Valley, 42.21953°N, 12.4175°E, 21 June 2020, leg. G. Tropea, 2 ♀♀, of which one subadult (GTC); Lago di Bracciano, Oriolo Romano (VT), 491 m, 6 March 2013, 3 ♂♂ (CNBFVR); VT, Lago di Vico, loc. Monte Venere, 701 m, 6 March 2013, 7 ♂♂, 7 ♀♀ (CNBFVR); Monti Cimini, Monte Venere verso E Cerreta 560–580 m, 24 July-23 August 1985, leg. S. Pedullà, M. Rellori, 6 ♂♂ (MZUR 92–95, 98, 99); near Calcata (VT), Treja’s valley, 28 July 2009, G. Tropea, 6 ♂♂, 2 ♀♀ (NCS); same data but 2 April 2012, G. Tropea, 1 ♂ (NCS); Oriolo Romano, 1 May 2010, G. Tropea, 1 ♂, 2 ♀♀ (NCS); Canale Monterano (TV), 22 May 2010, G. Tropea, 4 ♀♀ (NCS); Rio Fiume, Monti della Tolfa, 9 April 2012, G. Tropea, 6 ♂♂, 7 ♀♀ (NCS); same data but 22 May 2010, 4 ♀♀ (NCS); Gorge of the Biedano, Barbarano Romano (TV), 27 March 2010, G. Tropea, 1 ♂, 1 ♀ (NCS); Castel Giuliano (RM), 42.03381°N, 12.13032°E, 10 April 2010, G. Tropea, 1 ♂, 1 ♀ (NCS).

**Figure 19.**
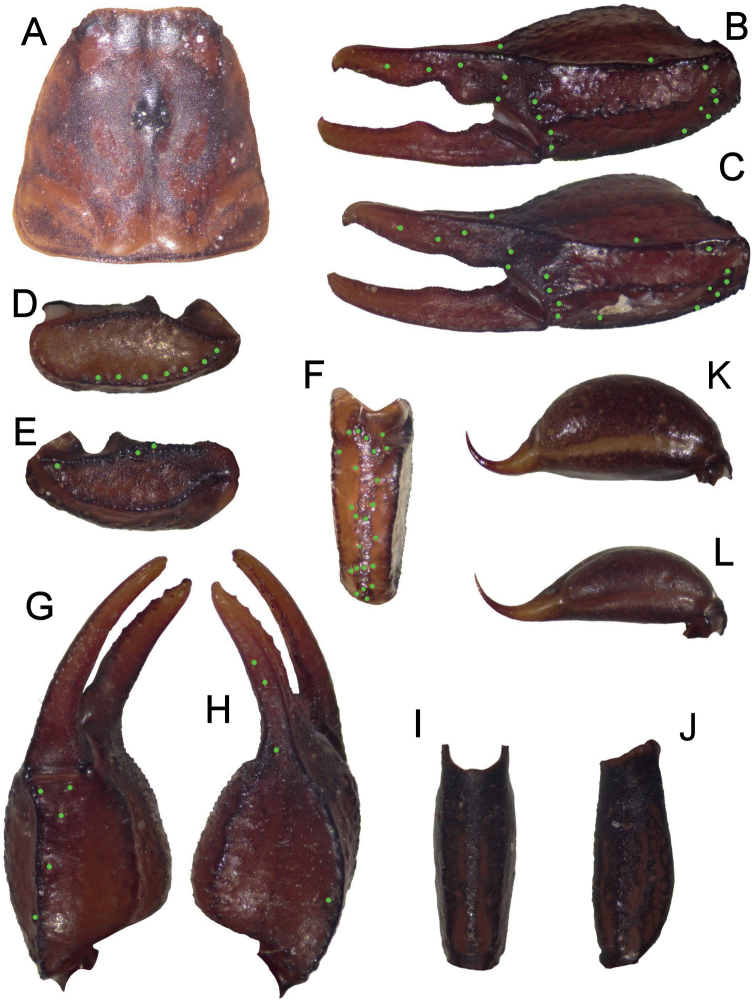
*Euscorpiustrejaensis* sp. nov. male holotype except Figs **C** and **N**, that are of a female paratype **A** carapace **B** external view of chela of adult male **C** external view of chela of adult female **D** ventral view of pedipalp patella **E** dorsal view of pedipalp patella **F** external view of pedipalp patella **G** ventral view of chela **H** dorsal view of chela **I** ventral view of metasomal segment V **J** lateral view of metasomal segment V **K** telson of adult male **L** telson of adult female.

##### Etymology.

The specific epithet is derived from Treja, the river that flows in the homonymous valley where the first specimens of *E.trejaensis* sp. nov. were collected.

**Figure 20.**
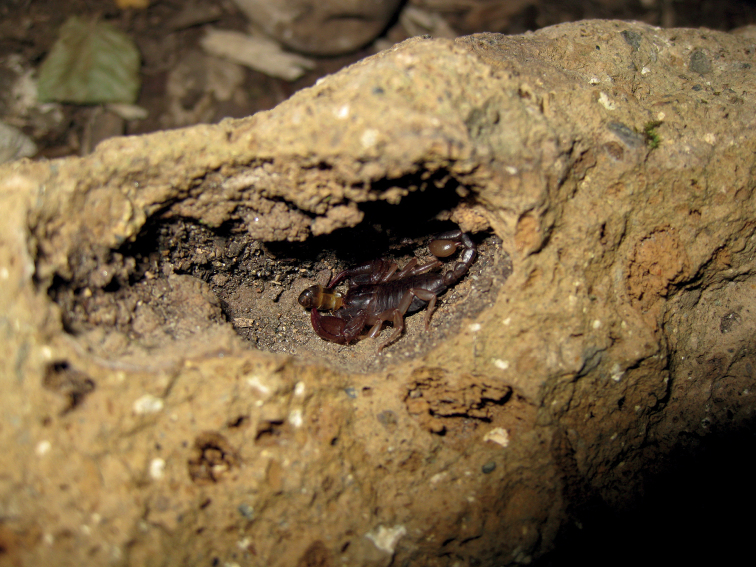
*Euscorpiustrejaensis* sp. nov. male photographed in nature feeding on an insect inside a small cavity of a tuff stone.

##### Geographic range.

Italy: Latium (right side of the Tiber River; Fig. 32).

**Figure 21.**
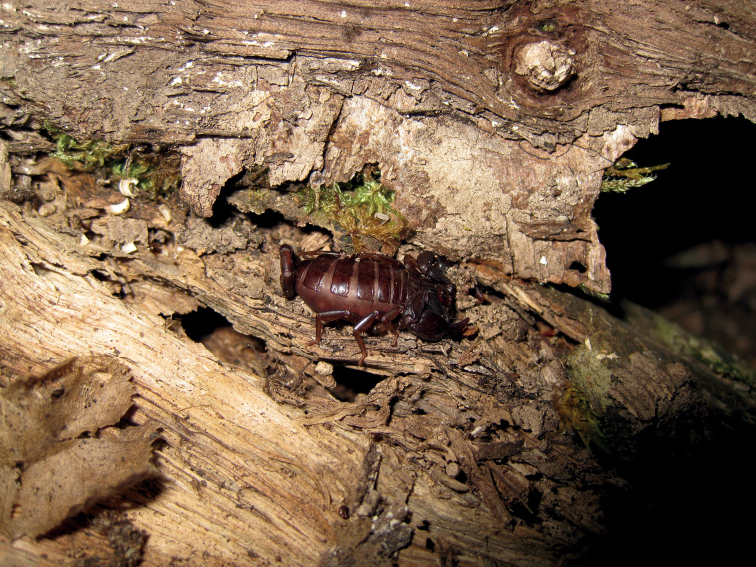
Pregnant *Euscorpiustrejaensis* sp. nov. found under the bark of a trunk.

##### Diagnosis.

A small *Euscorpius* species, total length 24–28 mm. Colour of adults mostly dark brown with darker marbling on most of the body, including chelicerae. The number of trichobothria on the pedipalp manus ventral surface is four (V_1–3_ + Et_1_). Trichobothria est and dsb on fixed finger are respectively located distally and proximally to the notch of the fixed finger. The number of trichobothria on the pedipalp patella ventral surface is usually seven and eight (seven in 34.07% of the pedipalps examined). The number of trichobothria on pedipalp patella external surface is usually: eb = 4, eb_a_ = 4, esb = 2, em = 4, est = 4, et = 6. Trichobothrium i of the femur is slightly proximal to or at the same level of d. The pectinal teeth number in males usually is eight (seven to nine) and in females usually is seven (six to eight). Dorsal patellar spur well developed. Femur is slightly shorter than the patella. Carapace approximately as long as wide, but it tends to be shorter than long in the females. Carinae V_1_ follows an external direction to the trichobothria Et_1_, without forming a Y-shape. Spinules on legs ending with a Y-shape. Ventrolateral and ventromedian carina on metasomal segment V well formed by small, spaced, slightly serrulated granules.

**Figure 22.**
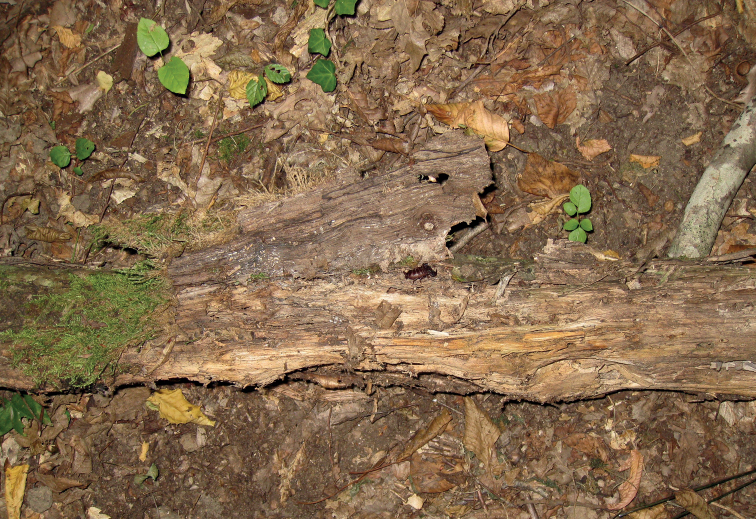
Barked trunk in which some specimens of *Euscorpiustrejaensis* sp. nov. were found.

##### Trichobothrial and pectinal teeth count variation.

The variation observed in 113 examined specimens (43 ♂♂ and 70 ♀♀) is given below (left/right asymmetry not specified).

Pectinal teeth in males (*n* = 86): 7/8 (7); 8/8 (28), 8/9 (5), 8/10 (1), 9/9 (2); in total, 7 in 8.14% (7), 8 in 80.23% (69), 9 in 10.46% (9) and 10 in 1.16% (1); mean = 8.05, SD = 0.48.

Pectinal teeth in females (*n* = 137): 6/6 (9), 6/7 (11), 7/7 (41), 7/8 (5), 8/? (1), 8/8 (1), 9/8 (1); in total, 6 in 21.17% (29), 7 in 71.53% (98), 8 in 6.57% (9), 9 in 0.73% (1); mean = 6.87, SD = 0.54.

Pedipalp patella trichobothria Pv (*n* = 226): 6/6 (1), 6/7 (4), 7/7 (22), 7/8 (29), 8/8 (52), 9/8 (3); in total, 6 in 2.65% (6), 7 in 34.07% (77), 8 in 61.95% (140), and 9 in 1.33% (3); mean = 7.62, SD = 0.56.

Pedipalp patella trichobothria Pe (*n* = 159): et = 5/4 (2), 5/5 (3), 4/6 (1), 5/6 (9), 6/? (1), 6/6 (60), 7/6 (1), 7/7 (3); in total, 4 in 1.89% (3), 5 in 10.69% (17), 6 in 83.02% (132) and 7 in 4.40% (7); mean = 5.90, SD = 0.47;

est = 3/3 (1), 4/? (1), 4/3 (2), 4/4 (79), 5/4 (1); em = 3/? (1), 3/3 (1), 3/4 (10), 4/4 (71), 5/4 (1); esb = 2/? (1), 1/2 (2), 2/2 (81); eb_a_ = 3/3 (1), 3/4 (6), 4/? (1), 4/4 (74), 4/5 (2); eb = 3/4 (1), 4/? (1), 4/4 (82).

##### Description of the male holotype.

***Colouration*.** A general dark brown base colour with more or less marked paler marbling or reticulation, reddish brown, in the less granulated areas, especially of the metasoma, legs, pedipalps and chelicerae; telson mostly dark brown with two ventrally longitudinal pale brown stripes and one for each side, with reddish brown distal part of the sting; ivory chelicerae with dark brown reticulation; chelae with fingers ranging from pale yellowish brown to dark reddish brown with dark blackish brown reticulation; legs with almost completely ivory tarsus, the basitarsus and tibia are mostly ivory, but with dark blackish brown marbling externally, almost ivory internally, the patella and femur are mostly dark with paler marbling externally, and mostly ivory internally; pectines and genital operculum whitish ivory; sternites range from almost completely black with pale spot on the most distal to very pale brownish at the most proximal.

**Figure 23.**
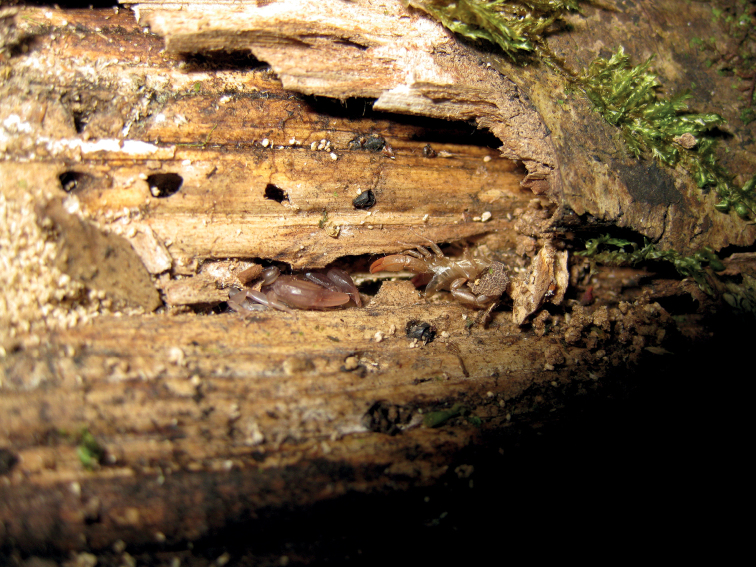
Specimen of *Euscorpiustrejaensis* sp. nov. which has recently carried out ecdysis under a bark.

***Carapace*.** Almost completely covered by a dense fine granulation; anterior edge is granulate; deep posterior lateral furrows; two pairs of lateral eyes, and a pair of median eyes; length from centre of median eyes to anterior margin is 40.98% of carapace length.

**Figure 24.**
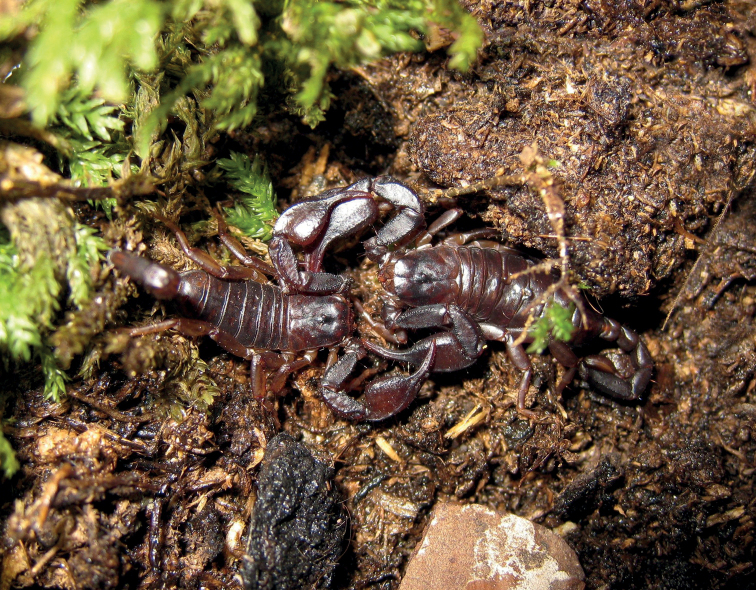
Beginning of mating of *Euscorpiustrejaensis* sp. nov., with the male grabbing the female’s chelae.

***Mesosoma*.** The tergites are densely covered with a fine granulation; sternites glossy and finely punctuated; small spiracles inclined to ~ 40° downward towards outside.

***Metasoma*.** Dorsal carinae on segments I–IV with spaced granules; ventrolateral carinae on segment I absent, on segment II and III smooth or obsolete, on segments IV, little marked with some small and spaced granule, with small slightly serrulated granules on segment V; ventromedian carinae absent on segment I–IV, on segment V it consists of small, slightly serrulated granules; dorsal and lateral intercarinal surfaces on segments I–IV are mostly finely granulated, especially on dark marbling, while the ventral surfaces are mostly smooth, the V segment is mostly finely granulated.

**Figure 25.**
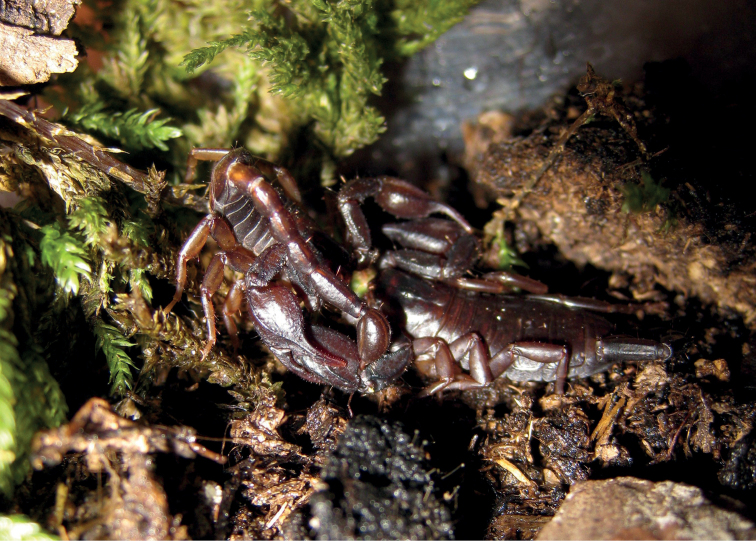
Lateral view of a male calming the female by stinging during mating.

***Telson*.** Vesicle with a few small granules, with ventral setae of different size, especially near the vesicle/aculeus juncture.

***Pectines*.** Teeth number 8/8; middle lamellae number 5/5; several microsetae on proximal area of teeth, marginal lamellae, and middle lamellae.

***Genital operculum*.** The genital operculum is formed by two longitudinally devised subtriangular sclerites with genital papillae protruding.

***Sternum*.** Pentagonal shape, type 2; slightly wider than long, with a deep posterior emargination.

***Pedipalps*.** Coxa and trochanter with tuberculated carinae. Femur: dorsal internal and external and ventral internal carinae tuberculated; irregular ventral external carinae formed by tubercles only on 1/3 or 1/2 of femur length; external median carinae formed by lightly serrulated tubercles; anterior median carinae formed by some spaced conical tubercles with three macrosetae; intercarinal spaces granulated. Patella: dorsal and ventral internal carinae tuberculated; ventral external carinae crenulated; dorsal external carinae slightly crenulated; intercarinal surfaces finely granulated, especially on the dark reticulations near the carinae. Dorsal patellar spur well developed. Chela: chelal carina D_1_ is distinct, strong, dark, and from smooth to slightly crenulated with a few tubercles proximally; D_4_ is dark with flat joined tubercles; V_1_ is distinct, strong, dark, from rough to crenulated, following an external direction to the trichobothria Et_1_; V_3_ is rounded with scattered granules; external carina granulated; intercarinal tegument granulated; the fixed and movable fingers with small, marked notch and lobe, respectively.

**Figure 26.**
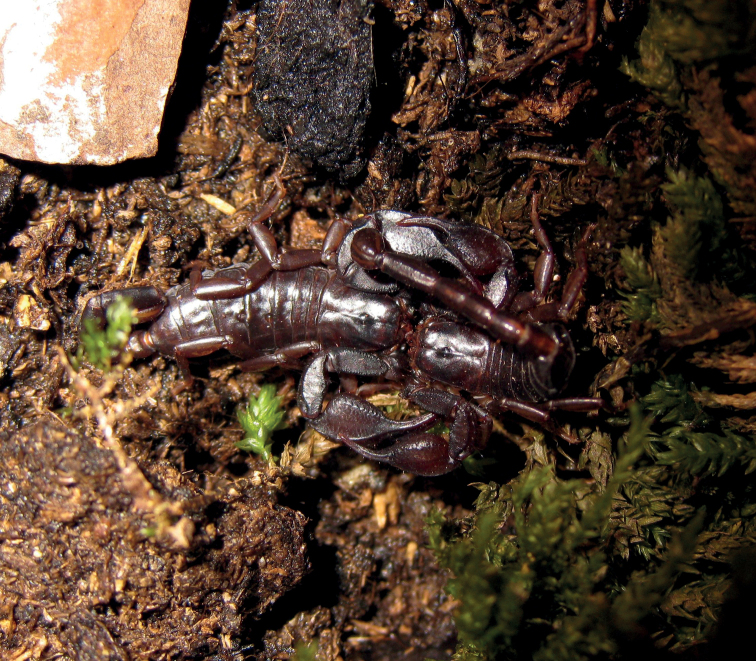
Dorsal view of a male calming the female by stinging during mating.

***Finger dentition*.** In the distalmost part a DD is present on the tip; MD is formed by very small denticles closely spaced, forming an approximately straight line, discontinued at level of the OD; fixed finger has 6/6 OD and 10/10 ID; movable finger has 8/8 OD and 13/11 ID.

***Trichobothria*.** Chela: trichobothria on the pedipalp manus ventral surface V = 3/3 (V_1–3_) + Et_1_ = 1/1; trichobothrium V_4_ situated on the external surface of the chela carina near the carina V_1_; trichobothrium ratio of et-est/est-dsb is ~ 1.43 and 1.25. Patella: Pv = 7/7; et = 5/6, est = 4/4, em = 4/4, esb = 2/2, eb_a_ = 4/4, eb = 4/4. Femur: trichobothrium d is slightly proximal to i, while trichobothrium e is well distal to both d and i, and situated on dorsal surface on dorsal external carina.

**Figure 27.**
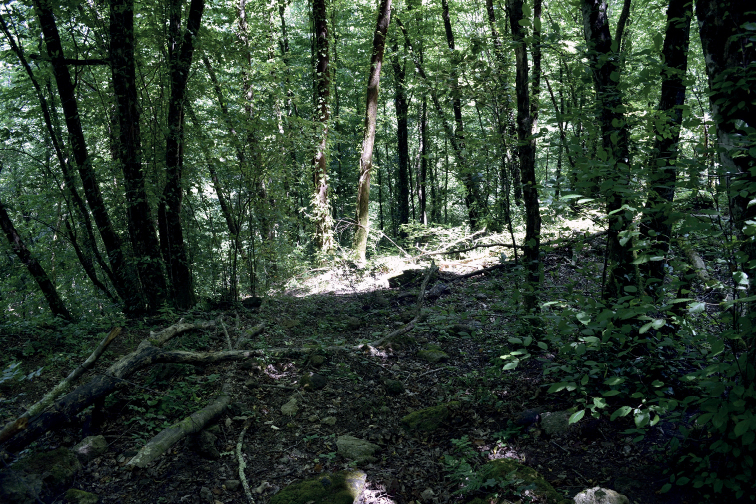
Example of the habitat of *Euscorpiustrejaensis* sp. nov.

***Legs*.** Two pedal spurs present; no tarsal spur; ventral row of tarsus with a total of 10/13 spinules on leg III, of increasing size from proximal to distal, ending with two spinules to form a Y-shape; three main flanking tarsal setae present. Tubercles present on ventral and dorsal surface of all leg femora.

***Chelicerae*.** Typical of the genus *Euscorpius*.

##### Description of the hemispermatophore.

Type A. It has a well-developed lamina tapered distally; well-developed basal constriction present; truncal flexure present; median projection with lde, ldi, and lb; internal projection distally with 5–7 tines in its crown. The number and the shape of tines of the crown varied between specimens and between the right and the left hemispermatophores.

##### Comments.

*Euscorpiustrejaensis* sp. nov. is geographically the closest species to *E.latinus* sp. nov., also part of the *E.concinnus* group, which seem to be divided from each other by the Tiber River. As mentioned above, this geographical proximity does not seem to result in a particular genetic relatedness. Indeed, according to the concatenated phylogenetic tree 16S + COI, *E.trejaensis* sp. nov. is paired with *E.stefaniae* sp. nov., with *E.niciensis* stat. nov. basal to them and between *E.latinus* sp. nov. and them. Regarding the divergence in 16S, between *E.trejaensis* sp. nov. and *E.latinus* sp. nov., it ranges from 2.7% to 3.1%, 3.1% with *E.stefaniae* sp. nov., and 2.7% with *E.concinnus* and *E.niciensis* stat. nov. Morphologically, like the other species of the *E.concinnus* group and the many cryptic species complex that have been described in recent years, *E.trejaensis* sp. nov. is difficult to distinguish without knowing its origin and having a good sampling size. As for the trichobothrial Pv values, we see that *E.trejaensis*, together with *E.latinus*, has the lowest average of Pv, ~ 7.60, having the percentage of Pv = 7 a little lower than in *E.latinus* sp. nov. (34.07% against 39.68%), but much higher than the other species treated here (2.78–13.33%); it has a percentage of *Pv* = 8 similar to *E.concinnus* and *E.latinus* sp. nov. (60.32–65.25%), much higher than *E.niciensis* stat. nov. (25% vs. 61.95%) and lower than *E.stefaniae* sp. nov. (81.67%). As for the Dp in males, *E.trejaensis* sp. nov. has the lowest average, the highest percentage of Dp = 8 and the lowest percentage of Dp = 9. These values are very different from those of *E.niciensis* stat. nov. and *E.stefaniae* sp. nov., and more similar to those of *E.concinnus* and *E.trejaensis* sp. nov. The Dp in females also reflects the same trend, albeit to a lesser extent. In fact, *E.trejaensis* sp. nov. has the lowest average and the highest percentage of Dp = 7 and 6, and the lowest percentage of Dp = 8.

The distribution of *Euscorpiustrejaensis* sp. nov. affects the central-north western part of Lazio, on the right side of the Tiber River. However, it must be ascertained whether its diffusion continues northward into Tuscany and Umbria. *Euscorpius.trejaensis* sp. nov. was found from 100 m a.s.l. on the Tolfa Mountains to 700 m a.s.l. on Mount Venere. This lower altitude is probably caused by the fact that this area of Lazio has no particularly high mountain formations, but mostly hills and low mountains. *Euscorpiustrejaensis* sp. nov. has always been found in natural areas, mostly in mesophilic forests, often with nearby streams, or in any case in very humid microhabitats. It showed mostly corticolous but also lapidicolous tendencies, having been found especially under the bark or cracks of fallen and rotting branches and trunks, or very damp, but also under stones, especially where there were few or no adequate branches. *Euscorpiustrejaensis* sp. nov. was found a few centimetres from *E.italicus* once in the type locality; however, despite having examined the areas several times over the years, *E.italicus* has no longer been found. Probably the latter prefer rural and less humid areas, unlike *E.trejaensis* sp. nov., so their meeting is infrequent.

*Euscorpiustrejaensis* sp. nov., like most species of *Euscorpius*, mate in spring and summer. The male grabs the female’s chelae and is in a constant state of alert and distrust (Fig. 24); the male stings the female between claw and patella of the pedipalp (Figs 25, 26), after which the female become calmer and cooperative. Thus begins a push and pull similar to a dance, but without the typical kiss of the scorpion (i.e., holding with chelicerae) observed in other species of scorpions (such as those belonging to the genus *Heterometrus* Ehrenberg, 1828), until the male finds a suitable surface to place the spermatophore and pulls the female until she goes over it, and the spermatophore fits into the genital operculum of female. Births usually take place in the summer of the following year, mostly in the months of July and August.

#### ﻿ Euscorpius niciensis

Taxon classificationAnimaliaScorpionesEuscorpiidae

(C.L. Koch, 1841) stat. nov.

9F6CE18C-812B-5A39-B523-C39A1DD7A850

**Figure 28.**
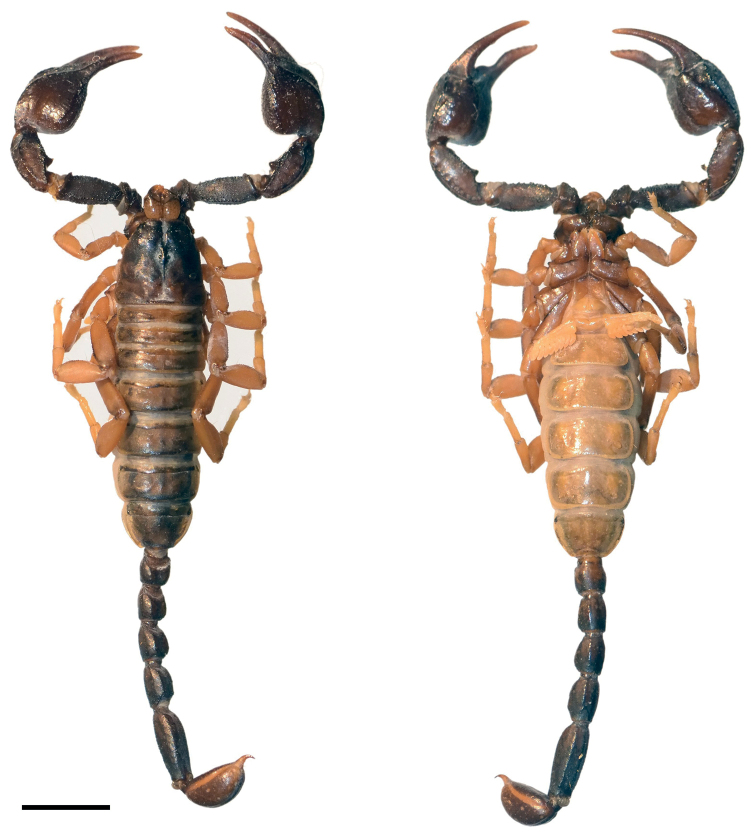
Dorsal and ventral view of *Euscorpiusniciensis* stat. nov. male neotype. Scale bar: 5.00 mm.

**Figure 29.**
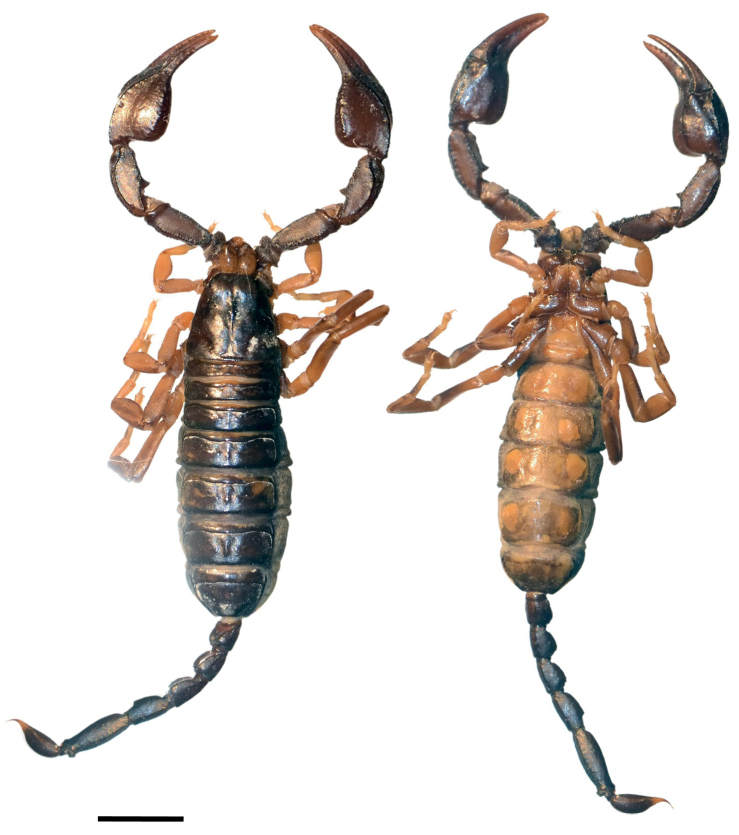
Dorsal and ventral view of *Euscorpiusniciensis* stat. nov. female topotype. Scale bar: 5.00 mm.

**Figure 30.**
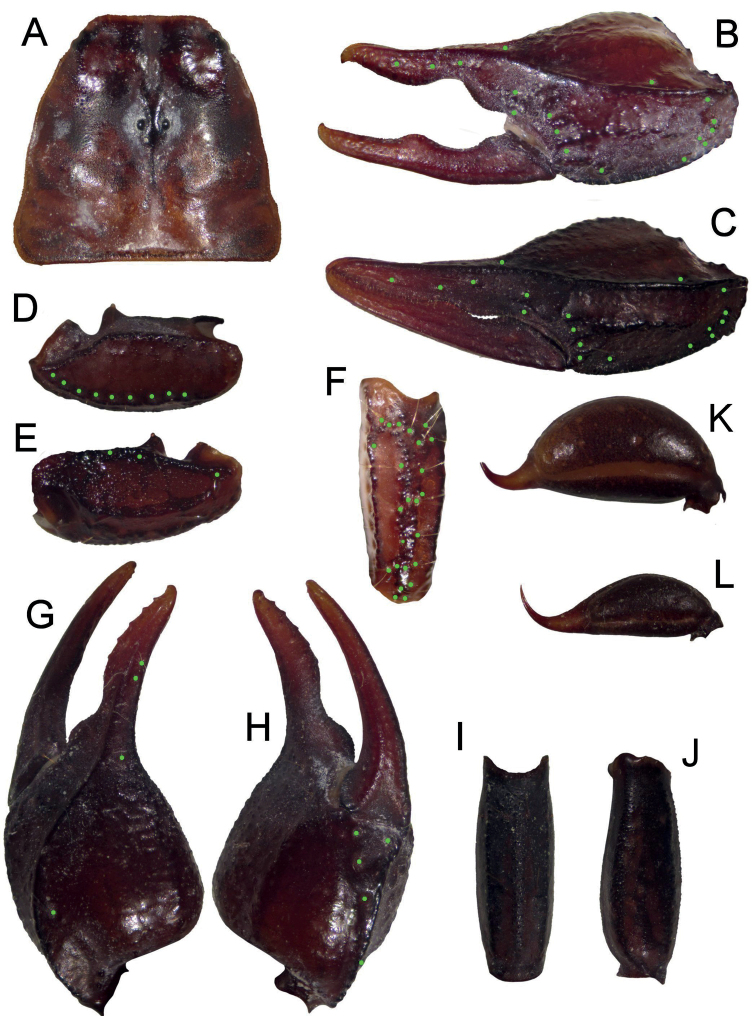
*Euscorpiusniciensis* stat. nov. male neotype except Figs **C** and **N**, that are of a female topotype. **A** carapace **B** external view of chela of adult male **C** external view of chela of adult female **D** ventral view of pedipalp patella **E** dorsal view of pedipalp patella **F** external view of pedipalp patella **G** dorsal view of chela **H** ventral view of chela **I** ventral view of metasomal segment V **J** lateral view of metasomal segment V **K** telson of adult male **L** telson of adult female.

Figs 28 , 29 , 30 , Table 1 , 3 , and 4

##### Type material.

***Holotype***: by C.L. Koch (1841), France, from the region/zone of Nice, is lost.

***Neotype***: ♂, France, Col de Braus (Nizza), 22 August 1975, leg. A. Valle, (MSNB 10234), here designated according to ICZN Article 75 as it is required for the purposes of clarifying the taxonomic status and type locality of a specific taxon.

##### Other examined specimens.

**France**: Same data as neotype but 4 ♂♂, 8 ♀♀ (MSNB 10232, 10233, 1035, 10605–10613); Colle dei Signori (Alpi Liguri), 2100 m, 1 August 1966, leg. A. Vigna, 1 ♂, 1 ♀ (MSNB 7375, 7376); Curbans (04), 20 August 2010, leg. E. Iorio, 1 ♂ (GTC); Entrevaux, 27 August 2018, leg. G. Ourliac, 2 ♂♂ (GTC); Gorges Daluis, 27 August 2018, leg. G. Ourliac, 3 ♂♂, 1 ♀♀ (GTC); Guillaumes, 25–27 August 2018, leg. G. Ourliac, 6 ♂♂, 2 ♀♀ (GTC); Guillaumes (06), 11 August 2018, leg. G. Ourliac, 1 ♂ (GTC); Meounes, Pes Montrieux, [Méounes-lès-Montrieux], 17 February 1973, leg. R. Bianchi et C. Fenaroli, 1 ♂ (MSNB 11354); Montrauraux (83), ZE1819, cheraie pierres, 19 May 2013, leg. E. Iorio, 1 ♀ (GTC); prov. Reotier (05), ZE RTE P3, 27 July 2010, leg. E. Iorio, 1 ♀ (GTC); St. Jeannet (6), 20 July 2018, 1 ♀ (GTC); **Italy**: Alassio (SAVONA), Valle ovest della Solva, 19 July, 1958, A. Valle, 7 ♀♀ (MSNB 832–835, 837–839); Andagna, Molini di Triora (IM), 30 May 1973, leg. R. Bianchi, 1 ♀ (MSNB 9823); Bardineto (SV), 26 August 1967, leg. A. Vigna, 1 ♂ (MSNB 7371); Castellaro (IM), 8 October 1969, leg. G.P. Rallo, 1 ♂ (MSNB 8032); Castellaro (IM), 8 October 1969, leg. Rallo, 1 ♂ (MSNB 8030); Celle Ligure (SV), 9 July 1956, leg. A Valle, 2 ♀♀ (MSNB 14, 16); Cesio (IM), 29 May 1973, leg. R. Bianchi, 1 ♂, 7 ♀♀ (MSNB 9802–9808, 9890); Chiusanico (IM), surroundings Di Torria, 7 March 1976, leg. M. Bologna, 1 ♂ (MSNB 10589); Chiusanico (IM), leg. 07 March 1976, leg. M. Bologna, 1 ♀ (MSNB 10588); Cosio d’Arroscia (IM), 27 June 1971, leg. R. Bianchi, 3 ♂♂, 5 ♀♀ (MSNB 8633–8641); Molini di Triora (IM), 30 May 1973, leg. R. Bianchi, 2 ♂♂, 1 ♀ (MSNB 9736, 9737, 9812); Monte Bardellini, (IM), 28 March 1970, leg. M. Bologna, 1 ♂, 1 ♀ (MSNB 9996, 9997); Sanremo (IM), 9 October 1969, leg. G.P. Rallo, 2 ♂♂, 1 ♀ (MSNB 8035–8037); Taggia (IM), 7 October 1969, leg. G.P. Rallo, 3 ♂♂, 1 ♀ (MSNB 8026–8028, 8034); Taggia (IM), 30 March 1970, leg. G.P. Rallo, 2 ♂♂, 3 ♀♀ (MSNB 8488–8492); Valdieri (CN), Andonno, 720 m, 26 August 1964, leg. A. Vigna, 1 ♀ (MSNB 4600); Ormea (CN), Ponte di Nava, 27 June 1971, leg. R. Bianchi, 2 ♂♂, 3 ♀♀ (MSNB 8642–8646).

##### Geographic range.

France and Italy (Fig. 32).

##### Diagnosis.

Medium *Euscorpius* species, total length 27–45 mm. Colour in adults usually brown to dark brown with darker brownish red pedipalps, and legs, chelicerae, and telson yellow or yellowish, but with sometimes legs and especially the telson brownish, with marbling especially on chelicerae, carapace, mesosoma, and metasoma. The number of trichobothria on the pedipalp manus ventral surface is four (*V*_1–3_ + *Et*_1_). Trichobothria est and dsb on fixed finger are respectively located distally and proximally to the notch of the fixed finger, although they can sometimes be only slightly distal or proximal. The number of trichobothria on the pedipalp patella ventral surface is usually nine and eight (mostly nine). The number of trichobothria on pedipalp patella external surface is usually: eb = 4, eb_a_ = 4, esb = 2, em = 4, est = 4, et = 6. The pectinal teeth number in males is usually nine and eight (nine in 60.26% and eight in 32.05%) and in females seven and eight (seven in 61.46% and eight in 33.33%). Dorsal patellar spur well developed. Femur approximately as long as patella but usually is slightly shorter. Carapace approximately as long as wide, but it usually is slightly wider than long. Carinae V_1_ follows an external direction to the trichobothria Et_1_, without forming a Y-shape. Spinules on legs ending with a Y-shape. Ventrolateral and ventromedian carina on metasomal segment V well formed by small serrulated granules.

##### Trichobothrial and pectinal teeth count variation.

The variation observed in 90 examined specimens (39 ♂♂ and 51 ♀♀) is given below.

Pectinal teeth in males (*n* = 78): 7/8 (1), 8/8 (8), 8/9 (8), 9/9 (18), 10/9 (3), 10/10 (1); in total, 7 in 1.28% (1), 8 in 32.05% (25), 9 in 60.26% (47), and 10 in 2.56% (5); mean = 8.72, SD = 0.60.

Pectinal teeth in females (*n* = 102): ?/? (3), 6/6 (1), 7/6 (2), 7/7 (24), 7/8 (9), 8/8 (11), 8/9 (1); in total, 6 in 4.17% (4), 7 in 61.46% (59) and 8 in 33.33% (32), 9 in 1.04% (1); mean = 7.31, SD = 0.57.

Pedipalp patella trichobothria Pv (*n* = 180): 7/7 (1), 7/8 (2), 8/8 (13), 8/9 (17), 9/7 (1), 9/9 (51), 10/9 (4), 10/10 (1); in total, 7 in 2.78% (5), 8 in 25% (45), 9 in 68.89% (124), and 10 in 3.33% (6); mean = 8.73, SD = 0.57.

Pedipalp patella trichobothria Pe (*n* = 180): et = 5/5 (4), 5/6 (3), 6/6 (68), 6/7 (6), 7/5 (1), 7/6 (2), 7/7 (6); in total, 5 in 6.67% (12), 6 in 81.67% (147) and 7 in 11.67% (21); mean = 6.05, SD = 0.43;

est = 3/4 (1), 4/4 (89); em = 3/3 (1), 4/3 (2), 4/4 (86), 5/4 (1); esb = 2/2 (90); eb_a_ = 4/4 (90); eb = 4/4 (90).

##### Description of the male neotype

**(MSNB 10234). *Colouration*.** A general reddish brown base colour, with blackish marbling or reticulation especially on the metasoma, mesosoma, carapace, pedipalp femur, patella, and chelicerae; telson mostly black with two ventrally longitudinal pale brown stripes and one for each side, with pale yellowish brown sting; pale brown chelicerae with dark brown reticulation; chelae dark reddish brown; legs orangish; pectines, genital operculum, and sternites yellowish.

***Carapace*.** Almost completely covered by dense fine granules, especially on the dark marbling. The granules in the lateral anterior part are larger; the anterior edge is straight and slightly granulate; deep posterior lateral furrows; two pairs of lateral eyes, and a pair of median eyes; length from centre of median eyes to anterior margin is 43.75% of carapace length.

***Mesosoma*.** Mostly of the tergites are very granulated, especially laterally; sternites glossy and punctuated; small spiracles inclined to ~ 45° downward towards outside.

***Metasoma*.** Dorsal carinae on segments I–IV granulated; ventrolateral carinae on segment I absent, on segments II and III smooth or obsolete, on segment IV with some granules, on segment V well marked with small serrulated granules; ventromedian carinae absent on segments I–IV, on segment V it consists of small, serrulated granules; intercarinal surfaces on segments I–IV are from finely granulated (e.g., dorsally) to smooth (e.g., ventrally), segment V is mostly granulated.

***Telson*.** Vesicle mostly smooth, with ventral setae of different size, especially near the vesicle/aculeus juncture.

***Pectines*.** Teeth number 9/8; middle lamellae number 5/5; several microsetae on proximal area of teeth, marginal lamellae, and middle lamellae.

***Genital operculum*.** The genital operculum is formed by two longitudinally divided subtriangular sclerites with genital papillae protruding.

***Sternum*.** Pentagonal in shape, type 2; wider than long, with a deep posterior emargination.

***Pedipalps*.** Coxa and trochanter with tuberculated carinae. Femur: dorsal internal and external and ventral internal carinae tuberculated; irregular ventral external carinae formed by tubercles just on 1/3 or 1/2 of femur length; external median carinae formed by lightly serrulated tubercles; anterior median carinae formed by some spaced conical tubercles with three macrosetae; intercarinal spaces granulated. Patella: dorsal and ventral internal carinae tuberculated; ventral external carinae crenulated; dorsal external carinae slightly crenulated to rough; intercarinal surfaces finely granulated, especially on the dark reticulations near the carinae. Dorsal patellar spur well developed. Chela: chelal carina D_1_ is distinct, strong, dark and smooth with a few flat tubercles proximally; D_4_ is rounded with a few spaced granules; V_1_ is distinct, strong, dark, from rough to crenulated, following an external direction to the trichobothria Et_1_; V_3_ is rounded with scattered granules; external carina granulated; intercarinal tegument granulated; the fixed and movable fingers with little marked notch and lobe, respectively.

***Finger dentition*.** In the most distal part is present a DD on the tip; MD is formed by very small denticles closely spaced forming an approximately straight line discontinued at level of the OD; fixed finger has 6/6 OD and 11/11 ID; movable finger has 8/7 OD and 13/13 ID.

***Trichobothria*.** Chela: trichobothria on the pedipalp manus ventral surface V = 3/3 (V_1–3_) + Et_1_ = 1/1; trichobothrium V_4_ is situated on the external surface of the chela, near the carina V_1_ well-spaced from it; trichobothrium ratio et-est/est-dsb is 1.31/1.17. Trichobothrium dsb is located slightly proximal at the centre of the notch. Patella: Pv = 9/9; et = 6/6, est = 4/4, em = 4/4, esb = 2/2, eb_a_ = 4/4, eb = 4/4. Femur: trichobothrium d is slightly proximal, almost on the same level, to i, while trichobothrium e is well distal to both d and i, and situated on dorsal surface on dorsal external carina.

***Legs*.** Two pedal spurs present; no tarsal spur; ventral row of tarsus with a total of eleven spinules, of increasing size from proximal to distal, ending with a Y-shape; three main flanking tarsal setae present. Tubercles present on ventral and dorsal surfaces of all leg femora, they are more marked and darker ventrally.

***Chelicerae*.** Typical of the genus *Euscorpius*.

##### Description of the hemispermatophore.

Type A. It has a well-developed lamina tapered distally; well-developed basal constriction present; truncal flexure present; median projection with lde, ldi, and lb, the latter with rounded tip; internal projection distally with 7–9 tines in its crown. The number and the shape of tines of the crown varied between specimens and between the right and the left hemispermatophores.

##### Comments.

*Euscorpiusniciensis* stat. nov. was described by C.L. Koch (1841) under the genus *Scorpius* from the region or zone of Nice, France. As explained by Tropea (2013, 2017), according to Koch the locality could be up to at least 50 km from Nice, so the exact type locality cannot be known. This species was described using only a few characters useful for identifying the species, and relying on a single specimen. In our study, several specimens from the surrounding areas of Nice and beyond were examined, and we found that those populations have significantly higher average and percentage values of Pv, Pe-et, and Dp than the closely related species, as can be seen in Tables 3 and 4. In fact, *E.niciensis* stat. nov. has an average Pv value of 8.73 with a percentage of 68.89% of Pv = 9, compared to an average ranging from 7.60 to 8.19 and a percentage of Pv = 9 ranging from 0% to 27.12% in the other species treated here. In addition, it is the only species (of the *E.concinnus* group) that, although with a limited percentage, showed a Pv = 10. The Pe-et percentages are also much higher than of other populations, with only some populations of *E.concinnus* approaching it. In fact, *E.niciensis* stat. nov. has a percentage et = 7 of 11.67%, compared to 1.23% of *E.latinus* sp. nov., 4.40% of *E.trejaensis* sp. nov., 5% of *E.stefaniae* sp. nov., and 9.32% of *E.concinnus*. To infer the phylogenetic trees, we have used only 16S (see Table 5). In the 16S+COI concatenated tree, it is placed between *E.latinus* sp. nov. and (*E.stefaniae* sp. nov. + *E.trejaensis* sp. nov.). Regarding the genetic divergence in 16S, *E.niciensis* stat. nov. is the most distant species from *E.stefaniae* sp. nov. with 3.8%, and 2.7% of divergence from all the other species, except *E.latinus* sp. nov. from Circeo, with which it has a divergence of 3.1%.

**Table 5.** DNA sequences used in phylogenetic analysis.

Species	Locality	Accession number and references	
		16S rRNA	COI mtDNA
*E.latinus* sp. nov. 1	Italy, Latium, Circeo	OL415488	OL415124
*E.latinus* sp. nov. 2	Italy, Latium, Lepini Mts.	OL415489	OL415125
*E.latinus* sp. nov. 3	Italy, Molise, Isernia Province	OL415490	OL415126
*E.latinus* sp. nov. 4	Italy, Latium, around Monterotondo (RM)	n.a.	OL415127
*E.latinus* sp. nov. 5	Italy, Latium, around Anticoli Corrado (RM)	n.a.	OL415128
*E.trejaensis* sp. nov. 1	Italy, Latium, around Calcata (VT)	OL415491	OL415129
*E.trejaensis* sp. nov. 2	Italy, Latium, around Calcata (VT)	n.a.	OL415130
*E.trejaensis* sp. nov. 3	Italy, Latium, Tolfa Mts., near Rio Fiume (RM)	n.a.	OL415131
*E.concinnus* 1	Italy, Tuscany, near Siena, type locality	DQ989940 (Salomone et al., 2007)	n.a.
*E.concinnus* 2	Italy, Tuscany, Livorno Hills, (LI)	OL415492	OL415132
*E.stefaniae* sp. nov.	Italy, Veneto, Padova Province, Euganean Hills	OL415493	OL415133
*E.niciensis* stat. nov.	France, Mathis, Maritime Alps	AJ389376 (Gantenbein et al., 1999)	n.a.
*E.tergestinus*	Croatia, Rab Island, Jurine, Banjol	KC215656 (Parmakelis et al., 2013)	KC215742 (Parmakelis et al., 2013)
*E.parthenopeius*	Italy, Campania, Naples	OL415494	OL415134
*E.aquilejensis*	Italy, Abruzzo, around Celano (AQ)	OL415495	OL415135
*A.germanus*	Italy, Trentino-Alto Adige	OL415496	OL415136
*T.flavicaudis*	Italy, Tuscany, Campiglia Marittima (LI)	OL415497	OL415137

With these findings, the validity of *E.niciensis* stat. nov. is unquestionable, even if it is not excluded that further taxonomic divisions exist in it, as well as for *E.concinnus*, which need to be confirmed with further studies.

**Figure 31.**
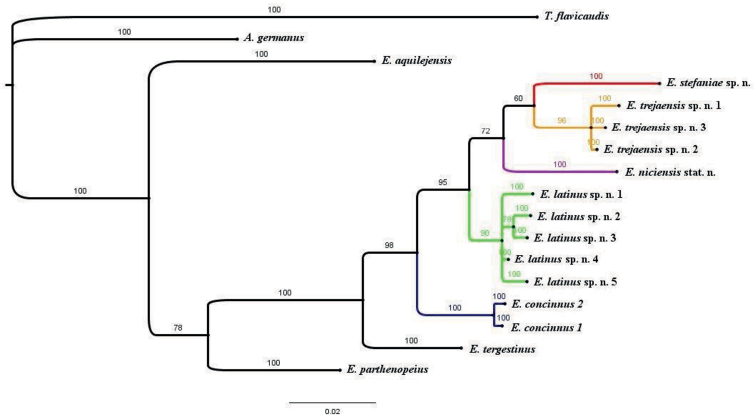
Phylogenetic tree based on the concatenated 16S rDNA and COX1 mtDNA loci. Posterior probabilities of nodes are shown (as percentages) on the branches. Different colours are used to indicate the newly described species. Scale bar corresponds to substitutions per site.

## ﻿Conclusions

In recent years, extensive morphological and molecular studies have indicated the great degree of speciation of the Euscorpiinae, as well as individual species usually having a limited range. This is in contrast with the idea that *E.concinnus* is a highly polymorphic species and the most widespread in Italy, as stated by Di Caporiacco (1950). The species of the *E.concinnus* complex cover an area that has been in the centre of extensive and repeated geological and climatic changes that have probably merged and divided the species’ populations on several occasions over time. These events have amplified the potential for speciation events and the presence of endemic species, but at the same time the delineation of species and inference of relationships has become a difficult task. Probably the populations of today are the result of expansions, extinctions, bottlenecks, and re-colonisations, perhaps several times due to the climatic changes in the Pleistocene. In this setting *E.concinnus* complex is one of the very complicated species group, and the reason that several studies have been performed (Di Caporiacco 1950; Fet and Soleglad 2002; Vignoli et al. 2005; Salomone et al. 2007; Tropea 2013) and are still ongoing (GT, in progress). For instance, further investigations are necessary regarding the populations assigned to *E.niciensis* stat. nov. and *E.concinnus* s. str. and s. l., that must involve a wider set of morphological features and molecular markers, with the inclusion of additional sampling locations. This could further increase the number of species included in this complex of *Euscorpius*.

**Figure 32.**
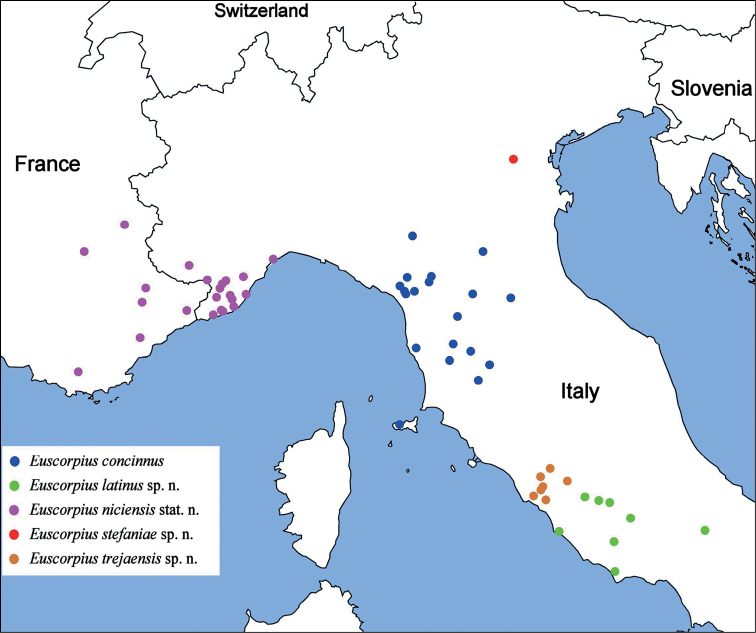
Maps of the known geographic ranges of the species treated herein.

## Supplementary Material

XML Treatment for Euscorpius concinnus

XML Treatment for Euscorpius latinus

XML Treatment for Euscorpius stefaniae

XML Treatment for Euscorpius trejaensis

XML Treatment for Euscorpius niciensis
